# High-resolution micro-CT for 3D infarct characterization and segmentation in mice stroke models

**DOI:** 10.1038/s41598-022-21494-9

**Published:** 2022-10-19

**Authors:** Raquel Pinto, Jan Matula, Maria Gomez-Lazaro, Mafalda Sousa, Andrea Lobo, Tomas Zikmund, Jozef Kaiser, João R. Gomes

**Affiliations:** 1 I3S – Instituto de Investigação e Inovação em Saúde, Universidade do Porto, Porto, Portugal; 2 Molecular Neurobiology, IBMC- Instituto de Biologia Molecular e Celular, Porto, Portugal; 3 Central European Institute of Technology, Brno University of Technology, CEITEC-BUT, Brno, Czech Republic; 4 INEB - Instituto de Engenharia Biomédica, Porto, Portugal; 5 Advanced Light Microscopy Unit, I3S – Instituto de Investigação e Inovação em Saúde, Universidade do Porto, Porto, Portugal; 6 Porto, Portugal

**Keywords:** Nervous system, Drug discovery, Neuroscience, Diseases of the nervous system, Regeneration and repair in the nervous system, Preclinical research, Translational research

## Abstract

Characterization of brain infarct lesions in rodent models of stroke is crucial to assess stroke pathophysiology and therapy outcome. Until recently, the analysis of brain lesions was performed using two techniques: (1) histological methods, such as TTC (Triphenyltetrazolium chloride), a time-consuming and inaccurate process; or (2) MRI imaging, a faster, 3D imaging method, that comes at a high cost. In the last decade, high-resolution micro-CT for 3D sample analysis turned into a simple, fast, and cheaper solution. Here, we successfully describe the application of brain contrasting agents (Osmium tetroxide and inorganic iodine) for high-resolution micro-CT imaging for fine location and quantification of ischemic lesion and edema in mouse preclinical stroke models. We used the intraluminal transient MCAO (Middle Cerebral Artery Occlusion) mouse stroke model to identify and quantify ischemic lesion and edema, and segment core and penumbra regions at different time points after ischemia, by manual and automatic methods. In the transient-ischemic-attack (TIA) mouse model, we can quantify striatal myelinated fibers degeneration. Of note, whole brain 3D reconstructions allow brain atlas co-registration, to identify the affected brain areas, and correlate them with functional impairment. This methodology proves to be a breakthrough in the field, by providing a precise and detailed assessment of stroke outcomes in preclinical animal studies.

## Introduction

Ischemic stroke is the leading cause of mortality and disability and the second leading cause of dementia in western countries^1^. The only treatment available focuses on reperfusion of the brain areas affected, via endovascular thrombectomy or blood clot-busting with tPA (tissue plasminogen activator)^2^. Despite important pre-clinical findings using stroke animal models, the lack of translation into clinical practice is pointed as the major factor that delays the implementation of new therapies^3^.

Rodents are the preferred animal model in nervous system research, due to their similarity to humans—in anatomy, biochemistry, and physiology-, and low maintenance and processing in the laboratory^4^. In rodents, stroke is most frequently modeled by transient occlusion of the middle cerebral artery (tMCAO), in which a coated monofilament is introduced into the vasculature to occlude the artery for the period intended by the researcher^1,5^. Occlusion of the middle cerebral artery (MCA) for small periods causes no neurologic deficit in mice, mimicking the clinical consequences of transient ischemic attacks (TIA)^6,7^; Occlusion for longer periods mimicks clinical cases of ischemic stroke, both in location and outcome. tMCAO causes a permanent lesion to the striatum (infarct core), irredeemably lost tissue, and penumbral characteristics to the surrounding cortex, a brain area that has reduced blood flow while preserving structural integrity^8,9^. Currently, the ischemic penumbra is the focus of many protective and therapeutic strategies, since this tissue keeps functional integrity and can be recovered^10^. Early after stroke onset, the penumbra is the largest part of the infarct lesion; and if no reperfusion occurs, it evolves into unrecoverable brain tissue (core). To study the specific location and temporal expansion of the penumbra, and its differentiation from the infract core, it is crucial to identify the salvageable brain tissue and to determine post-stroke recovery therapies^8,9^. In this study, we describe a novel methodology, using a combination of contrasting agents and micro-CT imaging, to evaluate the ischemic lesion and differentiate the core and penumbra.

Detailed characterization of a lesion in myelinated fibers (white matter) is critical to evaluate stroke outcome, since demyelination and axonal loss contribute to post-stroke disability. In this study, we describe a protocol to quantify microstructural differences and integrity of white fibers, and to quantify white matter volume vs grey matter^11,12^.

The characterization of stroke lesions in rodent models of stroke, traditionally relied on histological examination, which provides a fast (undetailed) way to calculate infarct volume from sectioned brains, and high-resolution images using contrast-enhancing staining, immunohistochemistry, or enzyme histochemistry^13^. However, these approaches are time-consuming, rely on quantification by estimation, and involve brain sectioning, which may lead to significant tissue damage and distortion, and the loss of part of the sample and of the brain 3D context^14–19^. It is possible to produce 3D assessments of brain injury, using both micro-MRI and X-ray micro-computed tomography (micro-CT)^20–22^. Micro-MRI allows in vivo brain scanning, longitudinal follow-up of individuals, and white/grey matter contrast. MRI is currently the only imaging technique that can distinguish lesion core and penumbra areas in both clinical and pre-clinical research^23–26^. However, MRI has a poor spatial resolution, in many cases with anisotropic voxels, which limits the ability to resolve brain fine structures, and complicates analysis and interpretation of the 3D data; MRI is also expensive and time-consuming. In our work, we show that micro-CT is a viable alternative to MRI, with higher spatial resolution, flexibility, and low cost. The 3D context, especially by the co-registration with a neuroanatomical segmented brain Atlas, together with behavioral assessments, contributes to produce more translatable data^18,20^. The main disadvantage of micro-CT is the low attenuation of X-rays by soft tissue^27^, which we overcome by using contrast agents to enhance soft-tissue X-ray contrast (such as osmium tetroxide (OsO_4_), inorganic iodine, phosphomolybdic acid (PMA) and phosphotungstic acid (PTA))^15,28^. These contrast agents can be applied to the brain either by perfusion or immersion^29^. The vasculature can be studied using polymerizing compounds or agents that penetrate the vessels lumen^30–35^, while neuronal tissue contrast can be achieved through immersion of the whole brain. Ionic contrast agents for the staining of soft tissues are used for brain imaging^36^ and whole-body^37^ of zebrafish, rodent embryos, brains, and other organs^27,28,37^. Some studies have gone further, staining whole mice brains to identify/quantify electrode lesions^38^, tumors^39^, malformations^40^, disease pathology in Alzheimer’s^41^, and even single neurons^42,43^. Nevertheless, the use of these stains to visualize and quantify cerebrovascular alterations^44^ and ischemic lesions^15,45–47^ in whole mouse brains are rarely described, and never with great tissue/lesion contrast.

While our described sample preparation and micro-CT measurement itself constitute major steps in the analysis, they are only the beginning of the whole pipeline. Image segmentation is the next crucial step to define structures, by assigning a class to each pixel/voxel of an image or volumetric data; it is particularly difficult for specific brain structures, due to the low contrast between different tissues and anatomical structures. Manual segmentation by a skilled operator to delineate the structures of interest by hand is still required to maintain accuracy and simplicity, but this is time-consuming and susceptible to operator bias, which is a significant bottleneck for the analysis pipeline, particularly in large-scale studies^15,41^. The focus is now shifting towards the development of semi-automatic and automatic tools to make image segmentation significantly faster. Deep learning and convolutional neural networks (CNNs) became were recently used for segmentation of the ant brain^48^, murine mineralized cartilage/bone^49^, and rabbit calcified knee cartilages^50^. While this segmentation provides morphological information about the object of interest, it lacks information on its function in the context of brain anatomy. This trouble can be solved by coupling micro-CT spatial information with an anatomical atlas to provide an accurate location of stroke-affected areas^51^.

In this study, we show how high-resolution micro-CT can improve stroke outcomes assessment in preclinical rodent models. We describe: (1) a simple and effective ex vivo brain staining method using two contrasting agents—Iodine and Osmium tetroxide-to originate images with high-quality, high-resolution, and neuroanatomical detail; (2) a novel methodology for precise quantification of 3D brain lesion, from brain edema to total infarct area, and core/penumbra segmentation; (3) the successful validation of this new approach compared to classical histological evaluation using TTC; (4) a semi-automatic method to evaluate white matter/grey matter changes and the integrity of myelinated fibers; (5) a novel automatic, deep-learning method for infarct and penumbra segmentation; and (6) the co-registration of 3D brain images with mouse atlas, allowing the identification of brain areas. We highlight the advantages of micro-CT imaging, and the complete methodology here described, as a relevant tool for the assessment of brain lesions in preclinical stroke studies.

## Results

### High-resolution micro-CT imaging allows the identification of distinct brain neuroanatomical areas

**Figure 1:**
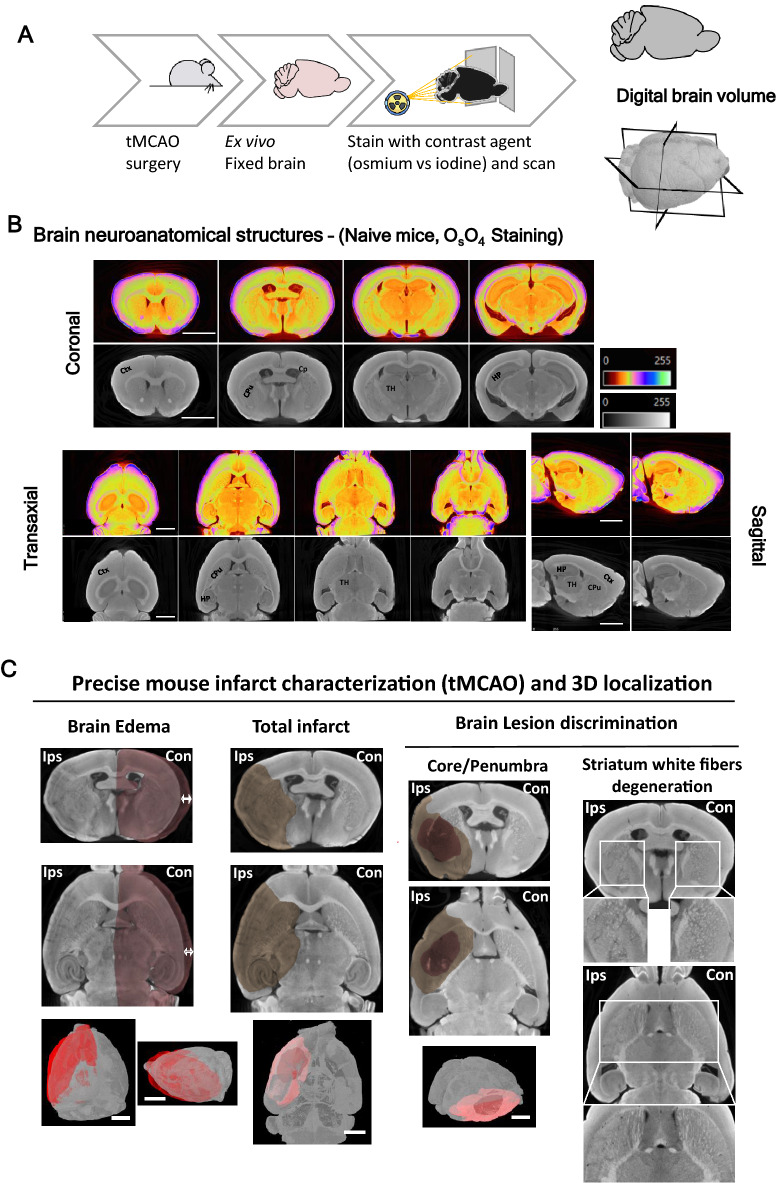
**Identification of brain neuroanatomical areas in physiological/pathological conditions (mouse stroke models) by high-resolution micro-CT imaging** (**A**) Overview of the technical steps required to prepare whole mice brains for micro-CT imaging. (**B**) Representative images of different brain projections (control, naive). Brains were stained with osmium tetroxide to reveal its neuroanatomical structures and differentiate white/grey matter. Regions such as cortex (Ctx), hippocampus and its sub-regions (HP, CA1-3, DG), choroid plexus (Cp), and striatum fibers in Caudate putamen (CPu) and thalamus (TH) are identified. (**C**) Representative 3D images of osmium staining to evaluate features induced by mouse stroke model (tMCAO) and TIA models, including brain edema (arrow indicates the brain swelling, comparison between contra/ipsilateral sides), total infarct area (dark area), core (highlighted darker area) and penumbra ((highlighted less dark area) discrimination, and striatum white fiber degeneration. Scale bar: 2 mm.

The methodology described in this work was developed to overcome the use of laborious histological techniques, or costly MRI apparatus, to characterize and quantify ischemic alterations in mice brains. Whole mice brains were subjected to different staining methodologies for soft tissues, previously described in the literature with limited success (low resolution and detail): osmium tetroxide, inorganic iodine, iohexol (Omnipaque), phosphomolybdic acid (PMA) and phosphotungstic acid (PTA). X-ray voltage and output current were fine-tuned to optimize volumetric reconstructions for each stain at isotropic resolutions and to allow histopathological interpretations (see materials and methods). The mice brains were stained horizontally mounted on the micro-CT apparatus, images were acquired, and brains were reconstructed to obtain a single 3D volume (Fig. 1A). Image dataset acquisitions took from 3 to 5 h, much faster than the acquisition by histological methods, which might take days. This is a major achievement for large-scale stroke studies that focus on the quantification of the lesion of a large number of mice brains, requiring high-throughput analysis.

Brains stained with osmium tetroxide showed high brain structural contrast in scans at resolutions of 3.5–4 µm voxel, allowing for a microscopic reconstruction of the whole-brain 3D volume (Fig. 1 B—coronal, sagittal, and transaxial views). Similar results were obtained with inorganic iodide (Supplementary Fig. 1). Importantly, the two stains produced complementary micro-CT contrasts: inorganic iodine is soluble in water, enhancing the contrast between grey and white matter; osmium is lipophilic, providing increased contrast to cell membranes and other lipid-rich structures, and creating a rather homogeneous contrast to all cells^21,25,37^. Both compounds allow a clear distinction of several brain structures such as the cortex (Ctx), striatum (caudate putamen- CPu), hypothalamus, thalamus (TH), and hippocampus (HP) (Fig. 1B and Supplementary Fig. 1). Importantly, Iodine presents lower toxicity than osmium (Table 1).

**Table 1:** **Comparison between osmium tetroxide vs inorganic iodine staining for whole-brain contrast enhancement**.

Contrasting agent properties	Osmium tetroxide	Inorganic Iodine
Binds lipophilic structures	Yes	No
Binds hydrophilic structures	No	Yes
Toxic	Yes	No
Expensive	Yes	No
Allows further histological processing	No	Yes
Penetration speed	Low	High
Brain shrinkage	No	Yes
Edema extent quantification	Yes	Yes
Lesion (core/penumbra) volume quantification	Yes	Yes
Striatum white matter degeneration quantification	Yes	Yes
Lesion (core/penumbra) volume quantification	Yes	Yes

In the case of iohexol, despite its ability to penetrate the whole sample, it conferred poor contrast to the different brain structures (Supplementary Fig. 2A). PTA and PMA were not able to stain the whole brain, since they did not penetrate completely in the tissue (Supplementary Fig. 2 B,C).

### Stroke-related lesions are observed on mouse brains using high-resolution micro-CT

**Figure 2:**
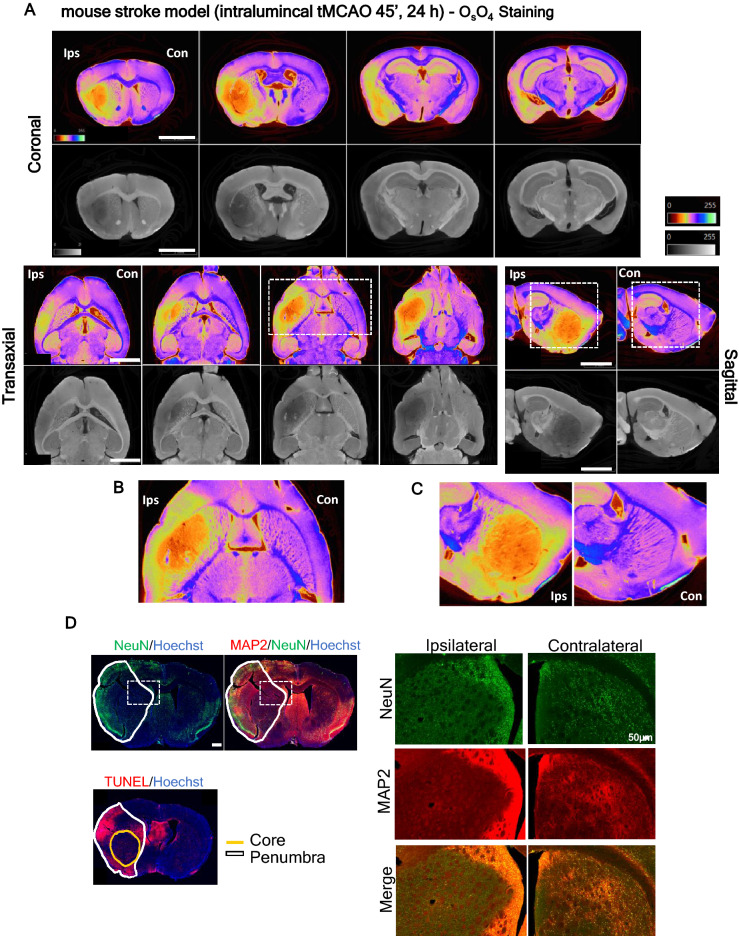
**Visualization of brain lesions in mouse stroke model (tMCAO) by high-resolution micro-CT imaging**. (**A**) Virtual slices (~ 4 µm voxels) at different orientations from a micro-CT scan of a mouse stroke brain stained with osmium tetroxide, showing total lesion and core (orange/darker area) and penumbra ((yellow/grey area around the core) differentiation. The below panel shows lesion detail in the (**B**) transaxial and (**C**) sagittal planes. Scale bar: 2 mm. (**D**) Representative brain slices immunolabeled with specific neuronal markers (NeuN and MAP2), an apoptosis marker -TUNEL, confirming that the lesion identified in micro-CT images is similar to the histologically defined lesion. Scale bar: 500 µm. The right side panel shows the lesion in detail, in the coronal plane. Scale bar: 50 µm.

Mouse brains subjected to ischemic insult were processed for tissue staining with either osmium tetroxide or inorganic iodine stains, revealing recognizable effects of the ischemic injury (Fig. 1C, 2A—osmium and Supplementary Fig. 1, iodine,): brain edema (Fig. 1C-white arrows indicate edema ipsilateral vs contralateral hemispheres), total infarct (Fig. 1C-dark area), differentiation between core and penumbra 24 h post-reperfusion (Fig. 1C-dark area- penumbra, darker area-core; Fig. 2A-C-yellow/grey around the core-penumbra, orange/dark grey-core; Fig. 2D-TUNEL/Hoechst staining- cell death in the core, Supplementary Video 1 and Supplementary Video 3), and tissue degeneration- striatal myelin degeneration and neuronal degeneration (Fig. 1C-white squares-striatum white fibers degeneration, ipsilateral vs contralateral hemispheres; Fig. 2D-NeuN and MAP2 staining- neuronal degeneration, ipsilateral vs contralateral hemispheres; Hoechst staining- cell death in core). Importantly, the total lesion and core/penumbra differentiation observed by micro-CT imaged brains (coronal sections) corresponds to the lesion boundaries observed by immunohistochemistry, indicated by cell death (TUNEL/Hoechst staining) and neuronal degeneration (NeuN, MAP2 labeling) (Fig. 2D and Supplementary Fig. 3).

Advantageously, iodine-stained brains could be used for further histological processing, since the tissue retains the structural components required for the recognition by the antibodies used in immunofluorescence protocols (Supplementary Fig. 3B: neurons stained with NeuN, dendrites with MAP2, nuclei with Hoechst; cell death assessed by TUNEL). This image acquisition workflow combines cellular data with anatomical and functional brain data, contributing to reduce the number of experimental animals. However, the dehydration steps performed in the iodine staining protocol induce brain shrinkage, with the final brain volume calculated as a third of the initial volume (Supplementary Fig. 3A). In the case of osmium staining, brains kept their original volume, but their further use for immunofluorescence analysis was prevented due to tissue mineralization^22,27,36,52^. Table 1 displays the advantages/disadvantages of the two contrasting agents (osmium tetroxide and inorganic iodine) for whole-brain contrast enhancement.

**Figure 3:**
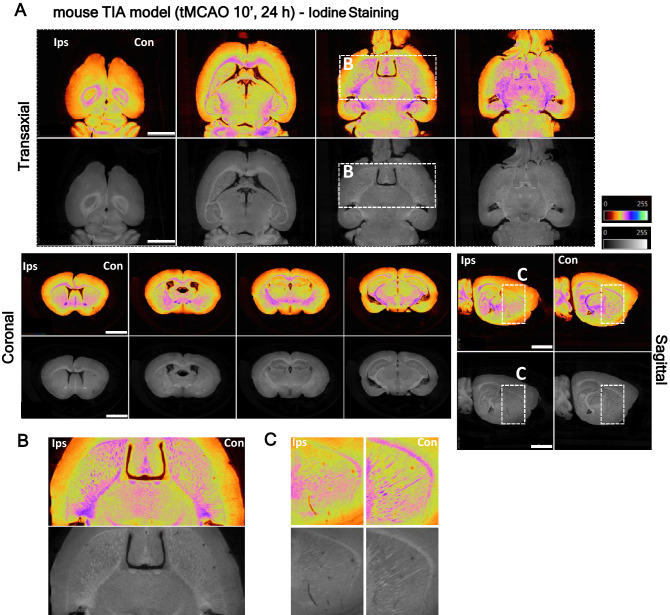
**Identification of striatum white fiber degeneration in transient ischemic attack (TIA) mouse model lesions by high-resolution micro-CT imaging.** (**A**) Virtual slices (~ 4 µm voxels) at different orientations from micro-CT scan of a TIA brain stained with inorganic iodine, showing striatum white fiber degeneration induced by 10 min MCA occlusion. (**B,C**) The below panel shows lesion detail in the transaxial and sagittal plane. Scale bar: 2 mm.

Additionally, striatum myelinated fiber degeneration was perceptible in the transient ischemic attack (TIA) model, with brains stained with either osmium or iodine, and corresponds to the total lesion and core/penumbra areas identified by micro-CT (Fig. 3, Supplementary Fig. 4, respectively-white stripes, pink/purple color; Supplementary Video 2). Micro-CT imaging allowed the detection of small lesions in myelinated fibres after the mouse TIA model. The contrast conferred by the stains (Osmium- Supplementary Fig. 4, Supplementary Video 2; Iodine—Fig. 3) enabled the visualization of white matter loss in the affected hemisphere’s striatum, when compared to the contralateral hemisphere, after 24 h of reperfusion. The white matter fiber tracts loss was uniform throughout the striatum, and not localized to regions within the ischemic injury, since this model does not present a visible infarct area (Fig. 3 and Supplementary Fig. 4). Thus, both osmium and iodine ex-vivo stained brains show clear stroke consequence demarcation that parallels histopathological-defined ischemia.

### High-resolution micro-CT allows fine manual quantification of mouse brain stroke lesion parameters

**Figure 4:**
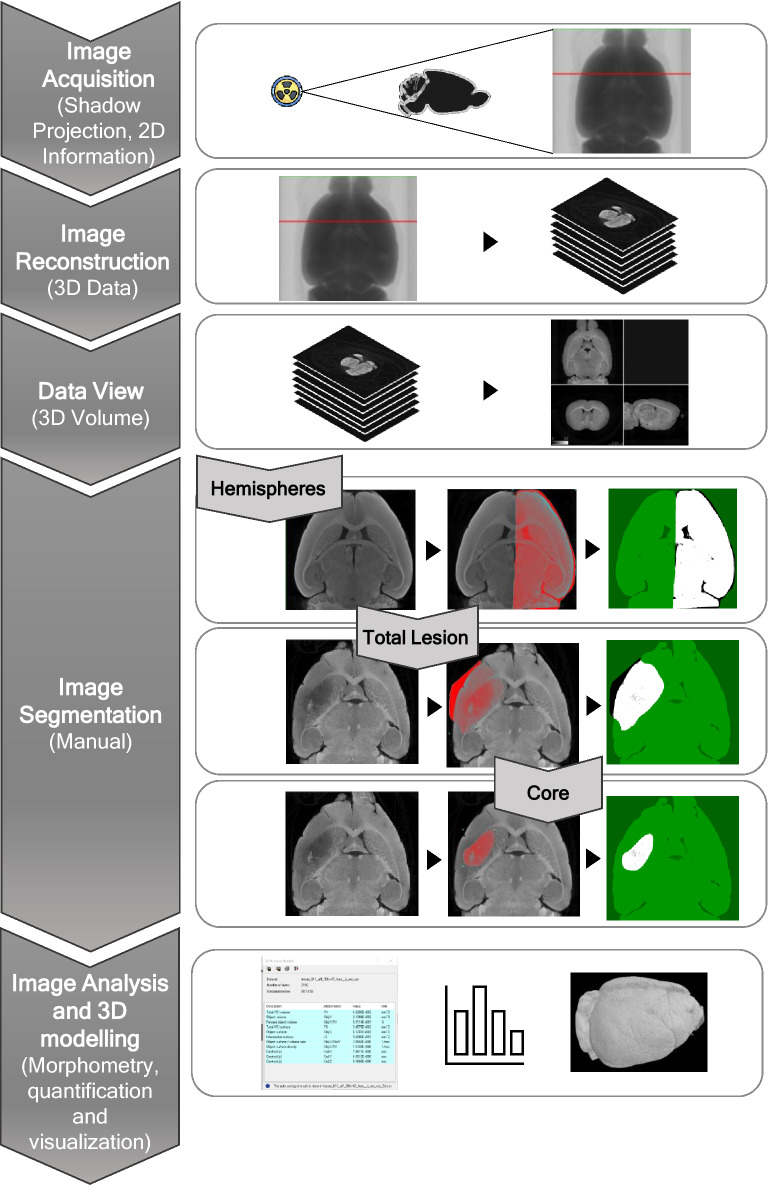
**Manual quantification workflow of different parameters in the ischemic mouse brain.** Representation of manual quantification procedure of mice brains subjected to stroke models and stained with a contrast agent. Images were reconstructed for 3D rendering using the NRecon software from BRUKER and visualized in the DataViewer BRUKER software. Analysis was performed in the CTAn software from BRUKER, in which hemispheres, total lesion, and core were manually delineated on the transaxial plane, and the volume occupied by the respective regions of interest was calculated.

**Figure 5:**
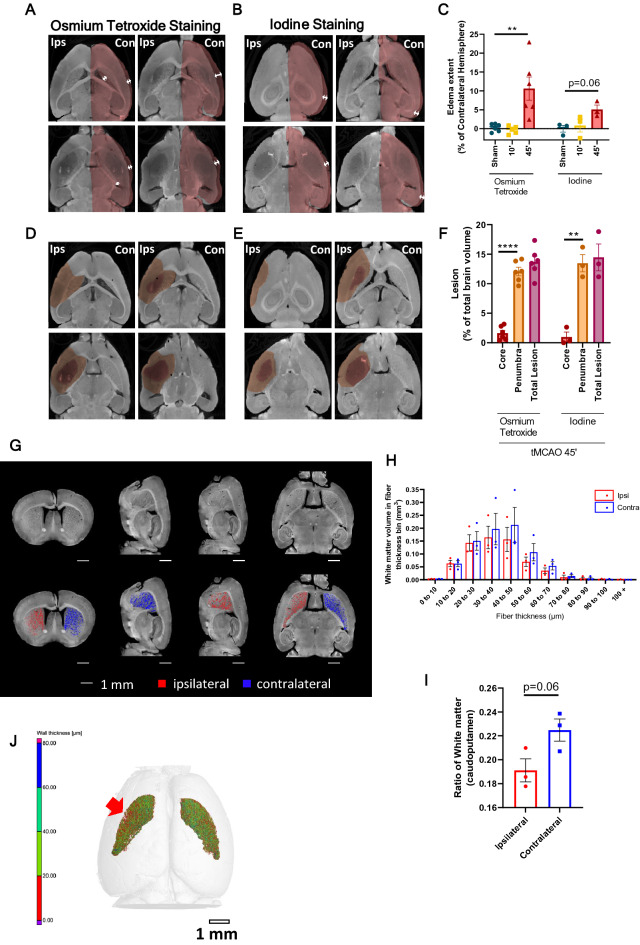
**Fine quantification and characterization of tMCAO (stroke and TIA-24 h) lesions in mice models by micro-CT imaging.** 2D slices in the transaxial plane showing: (**A**,**B**) edema (arrows indicate the brain swelling comparing contra/ipsilateral sides), (**D,E**) lesion penumbra (highlighted less dark area) /core (highlighted darker area), using osmium tetroxide (**A,D**) or inorganic iodine (**B,E**). (**C**)** Stroke** (tMCAO—24 h) induced brain edema extent, quantified using the manually obtained volumes for each brain hemisphere in the micro-CT images. (Osmium tetroxide: sham, n = 6; 10-min, n = 5; 45-min, n = 7, p = 0.0028 sham vs tMCAO 45 min. Inorganic iodine: sham, n = 3; 10-min, n = 3; 45-min, n = 3, p = 0.062 sham vs tMCAO 45 min). (**F**) Edema-corrected lesion volume (with discrimination between core and penumbra), as quantified using the edema extent and the total lesion/core volumes, manually segmented (Osmium tetroxide, tMCAO 45-min, n = 6, *p* < 0.0001; inorganic iodine, tMCAO 45-min, n = 3, *p* = 0.0017). Statistical analysis was performed using one-way ANOVA, followed by Dunnet’s or Sidak’s multiple comparison tests. **(G)** Representative images of iodine-stained brains from the TIA mouse model show white matter contrast at arbitrary orientation and white matter degeneration in the caudate putamen (striatum region) in the ipsilateral hemisphere. **(H)** Semi-automatic quantification of white matter volume in different individual fiber thicknesses, in both hemispheres (n = 3, A-C). (**I**) The ratio of caudate -putamen volume occupied by white matter in each hemisphere (ipsilateral vs. contralateral hemisphere), n = 3. (**J**) Heat map of striatum white matter wall thickness, as quantified and segmented by the semi-automatic method, the red arrow shows an area with a significant decrease in the fiber thickness in the ipsilateral hemisphere.

The detection of subtle differences in infarct volume is critical to evaluate the success of stroke therapy. By optimizing contrast-enhanced micro-CT imaging (osmium or iodine), the micro-CT proprietary software from BRUKER, CTAn, was used to manually delineate the mouse brain hemispheres, and the ischemic lesions (24 h), in the virtual slices (Fig. 4). The selected brain regions were used to calculate: edema extent (as % contralateral hemisphere) (Fig. 5A–C), total corrected lesion volume (% of total brain volume) (Fig. 5D–F), and discriminate ischemic core from penumbra (Fig. 5D–F). Osmium tetroxide-stained brains from mice subjected to the stroke tMCAO model (45-min occlusion, 24 h recovery) displayed edema that induced an increase in the ipsilateral hemisphere size by 10.6% ± 3%, when compared to the contralateral hemisphere (Fig. 5A–C, p = 0.0028); and exhibited brain lesions that occupied 13.7% ± 1% of the total brain volume (penumbra: 12.07% ± 0.7% and core: 1.6% ± 0.5%, *p* < 0.0001) (Fig. 5D–F). Iodine-stained brains from mice subjected to the same stroke model displayed edema–induced increase in the ipsilateral hemisphere size of 5.1% ± 2% (Fig. 5A–C, *p* = 0.062), and a total lesion that occupied 14.5% ± 2% of total brain volume (penumbra: 13.5% ± 1%, core: 0.9% ± 0.8%, *p* = 0.0017) (Fig. 5D–F). This approach offers several advantages over classical histological methods: the area occupied by the ventricles can be included/excluded in the calculations, depending on edema extent; and the brain midline, which shifts due to edema, can be adjusted for fine brain area selection. This new methodology was used to evaluate the time-dependent increase in infarct volume in the cortex/striatum, from 3 h to 72 h, and at 7 days post-reperfusion, and an edema-induced increase in the volume of the ipsilateral hemisphere in stroke and TIA mice models (Supplementary Fig. 5).

### Deep learning enables automatic lesion segmentation in micro-CT images

To improve reproducibility, high-throughput quantification, and reduce operator bias, we designed automatic segmentation and quantification processes to evaluate stroke lesions and white matter degeneration in brain micro-CT scans. This is a difficult task due to the low contrast between the lesion and healthy tissue, but iodine-stain of mice brain overcome that obstacle, by increasing the signal-to-noise ratio, allowing the segmentation of total lesion and discrimination of core and penumbra; and conferring contrast to white matter, for the evaluation of stroke-induced white matter degeneration.

**Figure 6:**
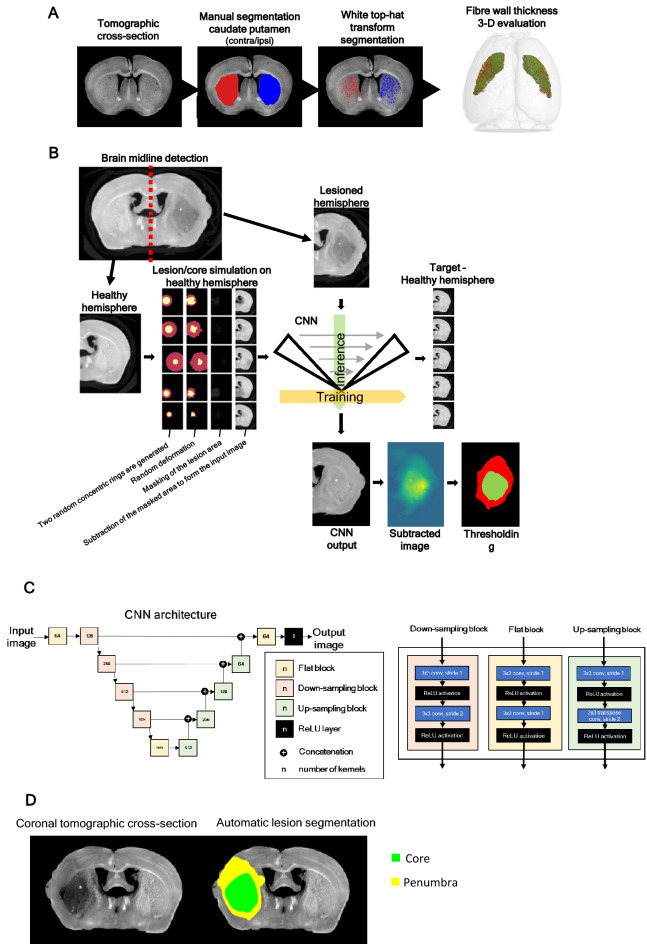
**Deep learning methods for semi-automatic evaluation of the white fiber degradation in the TIA model and automatic infarct lesion segmentation, in the stroke model.** In (**A**), we utilized a combination of manual segmentation of caudate putamen and automatic segmentation of white matter fibers via white top-hat transform. The thickness of the fibers was evaluated using the software VG Studio MAX 3.4 (VOLUME GRAPHICS GmbH, Heidelberg, Germany). In (**B**)**,** an automatic deep learning-based segmentation workflow is proposed for the segmentation of the lesion penumbra and core. (**C**) The convolutional neural network (CNN) is utilized for automatic lesion detection. (**D**) Representative images of automatic lesion segmentation in the iodine-stained brain of mouse stroke model (tMCAO 45 min).

We developed a semi-automatic method to quantify white matter, using brains from mice subjected to transient ischemic attack (TIA), and stained with iodine. Briefly, we manually segmented the caudate putamen (striatum) in high-resolution micro-CT images, applied a white-top threshold (Figs. 5G, 6A), and quantified white matter 3D fibbers (Fig. 5H-J). Our data reveals white matter degeneration induced by TIA: after 10-min ischemia, white matter fibers on the ipsilateral hemisphere occupied less 14% of the caudate putamen volume when compared with white matter fibers in the contralateral hemisphere (Fig. 5I), probably due to the decrease of fiber wall thickness (Fig. 5H).

For the analysis of stroke lesion and discrimination of core and penumbra, we applied an automatic deep learning-based segmentation and quantification (Fig. 6B–D). The method is based on training a convolutional neural network (CNN) to transform a lesioned brain hemisphere into an approximation of a healthy brain hemisphere, by randomly simulating the lesion in the available micro-CT cross-sections of healthy brain hemispheres. By subtracting the original and transformed image, and by subsequent thresholding the image obtained image, we produce segmentation masks of the whole lesion and core (Fig. 6B–D, Supplementary Fig. 6). The results of this automatic quantification in iodine-stained brains of the tMCAO model are shown in Table 2, and indicate that total lesion volume is 20.33 ± 4.48 mm^3^, and the core volume is 2.36 ± 1.56 mm^3^. These data are very similar to the data obtained by manual quantification -a total lesion volume of 21.63 ± 4.75 mm^3^ and a core volume of 3.10 ± 1.39 mm^3^.

**Table 2:** **Results of automatic quantification based on deep-learning vs manual segmentation of iodine-stained tMCAO brains.**

	Automatically segmented lesion volume (mm^3^)		Manually segmented lesion volume (mm^3^)	
	Total Lesion	Core	Total Lesion	Core
Sample 1	16.8906	3.7642	20.2020	5.0047
Sample 2	17.4301	3.1330	16.6580	2.5465
Sample 3	26.6595	0.1852	28.0330	1.7491

Taken together, these results indicate that high-resolution ex-vivo micro-CT (contrast-enhanced) offers several advantages to mouse preclinical stroke studies, compared to traditional histological methods: reduces the time for image analysis, reduces operator workload, and increases the outcomes obtained.

### Comparison of micro-CT imaging of mouse infarct/edema with histology staining

**Figure 7:**
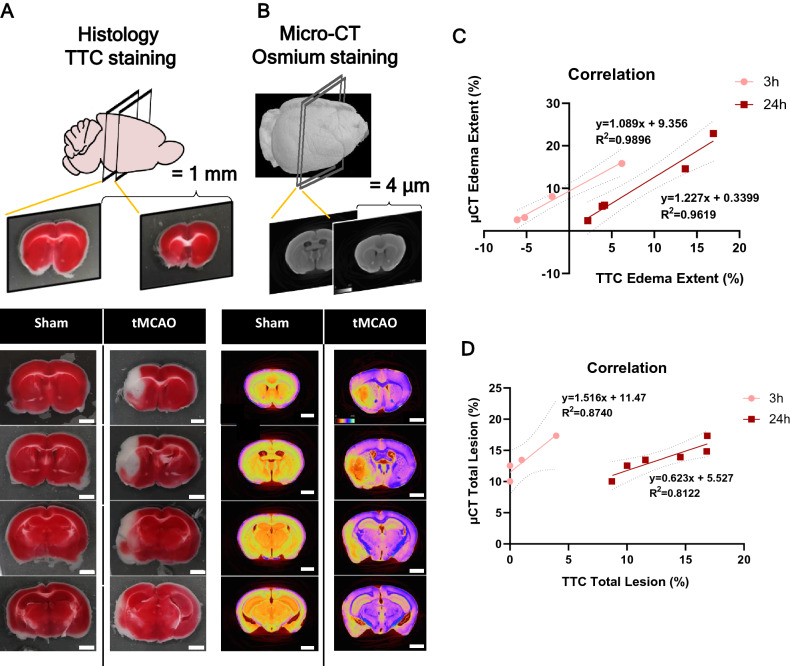
**Correlation between micro-CT imaging and the TTC method, for infarct and edema quantification.** (**A**) Representative histological cross-sections of TTC stained mice brain slices of sham and tMCAO 45 min animals, 24 h after reperfusion. (**B**) Representative 3D micro-CT volume renders cross-sectioning of both sham and tMCAO 45-min mice brains, 24 h after reperfusion. (**C**) Linear regression for comparison of edema extent (%) values measured by histology and micro-CT methods, at 3 and 24 h post-reperfusion. With the intercept fixed at zero, there is a significant correlation between the two measurement techniques (3 h after reperfusion: *p* = 0.0052, R^2^ = 0.9896; micro-CT brains, n = 4; TTC brains n = 4; 24 h after reperfusion: *p* = 0.0032, R^2^ = 0.9619; micro-CT brains, n = 4; TTC staining, n = 8). (**D**) Linear regression for comparison of the total lesion (%) values measured by histology and micro-CT, at 3 h and 24 h post-reperfusion. With the intercept fixed at zero, there was significant correlation between the two measurement techniques at 24-h post-reperfusion (3 h: *p* = 0.0651, R^2^ = 0.8740; 24 h: *p* = 0.014153, R^2^ = 0.8122; 3h: micro-CT, n = 4; TTC, n = 4. 24-h: micro-CT, n = 6, TTC, n = 6).

We compared the increase in brain volume caused by edema, lesion volumes, and core/penumbra areas quantified in micro-CT 3D reconstructed datasets (stained with osmium), with the histological Triphenyl tetrazolium chloride (TTC) method (Fig. 7A,B). TTC staining is the most used method to detect and quantify infarcts in rodents stroke models, due to the facility of the process^53^. This method is based on TTC reduction by mitochondrial oxidative enzymes, allowing the distinction between live and dead cells: if cells are alive, reduction of TTC occurs and cells will appear red, if cells are dead TTC is not reduced and cells appear white. However, this method poses important limitations: it can only be performed in fresh thick tissue slices (losing fine infarct localization); it only accounts for healthy mitochondria, meaning it can only be used in the first 24 h, before the infiltration of inflammatory cells that contain functional mitochondria; and does not address other stroke effects, such as white matter degeneration^54^. When comparing the edema extent and total lesion volume in sham vs tMCAO animals at 3 h and 24 h after reperfusion, micro-CT and TTC presented positive correlation indexes for edema of 0.98 (3 h, *p* = 0.0052) and 0.96 (24 h, *p* = 0.0032), and for total lesion volume of 0.87 (3 h, *p* = 0.0651) and 0.81 (24 h, *p* = 0014) (Fig. 7C,D). This data demonstrates that the lesion area calculated using the virtual slices obtained by micro-CT is in close agreement with the lesion area quantified by histological sectioning, confirming that the described demarcation corresponds to the ischemic infarction area. Hence, the novel methodology we propose can be used to accurately determine infarct volume in rodent stroke models.

### High resolution micro-CT imaging allows data co-registration with mouse brain atlas to identify specific brain regions

**Figure 8:**
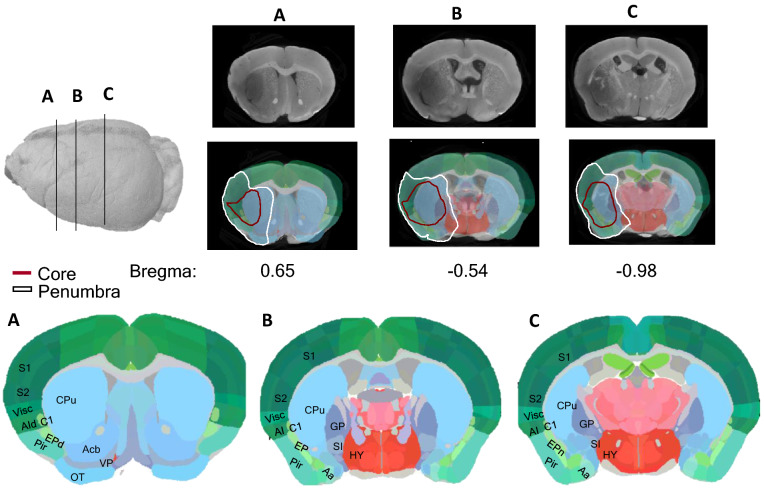
**Identification of brain areas by co-registration of micro-CT images with Allen Mouse Brain Atlas, using QuickNII.** JPEG images of the whole 3D volume of a representative tMCAO 45-min (24 h after reperfusion) mouse brain were uploaded to QuickNII software, and every 10 sections were aligned with the representative brain atlas. The brain structures affected by stroke were assessed and identified according to Table 3.

Reporting the location of the lesion, its extent, and how consistent these results are among different test subjects is important in preclinical stroke studies. Additionally, lesion registration to a specific sub-anatomical brain structure can be linked to potential functional consequences^18^. We took advantage of the serial section aligner QuickNII^®^ software that includes the mouse (C56BL7) anatomical brain areas identified and annotated according to the Allen Mouse Brain Atlas^55,56^, to associate brain section images acquired with high-resolution micro-CT to the corresponding brain region. We aligned the images of a stroke model, 45 min tMCAO mice model (24 h reperfusion), with the serial section aligner QuickNII^®^ tool (Fig. 8A–C), and identified the infarct affected anatomical areas^55,56^ (Table 2). We observed that the most affected area was the caudate putamen, which was altered after ischemic periods as shorter as 10 min, and several sub-regions of the cortex, involved in motor, sensory and associative circuits, such as the primary somatosensory, gustatory, agranular insular and the piriform areas.

**Table 3:** **Nomenclature of the brain regions affected by ischemia and respective abbreviations.**

Aa	Amygdalae
Acb	Accumbens nucleus
AI	Agranular insular area
C1	Claustrum
CPu	Caudoputamen
EP	Endopiriform nucleus
GP	Globus pallidus
HY	Hypothalamus
OT	Olfactory tubercle
Pir	Piriform area
S1	Primary somatosensory area
S2	Supplemental somatosensory area
SI	Substantia innominate
Visc	Visceral area

## Discussion

In this work, we report for the first time micro-CT imaging as an accurate and reliable technique for the detection and fine quantification of ex vivo brain ischemic lesions, and for the assessment of white matter degeneration, in rodent models of stroke.

The visualization of the brain’s internal structures by high-resolution CT imaging is possible by using a contrast agent. In the literature, several heavy-metal stains are commonly reported for contrast enhancement of soft tissues. So, we used osmium tetroxide, inorganic iodine, iohexol, PMA, and PTA to stain mouse brains, and analyzed their ability to yield anatomical contrast and identify established brain ischemic consequences in mouse models. Staining with osmium tetroxide and inorganic iodine yielded the best anatomical contrast, in comparison to the other substances tested (iohexol, PTA, and PMA). Similar staining protocols for PTA and PMA were used by Descamps et al^15^ to stain whole mouse embryos, however in our study, these compounds did not diffuse completely into the mouse brain, probably due to its big size. Dobrivojevic et al^57^ used iohexol to identify an ischemic lesion in a tMCAO mice model, but their data displays low contrast on brain anatomic structures and the ischemic lesion is very difficult to identify. As reported by Masis, et al^21^ and Zikmundet al^15^, osmium tetroxide and inorganic iodine staining protocols, respectively, resulted in contrast enhancement of the brain anatomy: they allowed visualization of ischemic degeneration, the definition of the lesion area and edema, the distinction between core and penumbra, and white matter degeneration. Since iodine binds to glycogen molecules, the contrast conferred by this agent reflects the bulk of water in the brain tissue, and allows the discrimination of brain structures due to high white and grey matter contrast^38^. Osmium binds mainly to phospholipids and proteins, and reflects cellular density, being powerful to demarcate most of the anatomical brain features^25^. Both stains allowed the visualization, manual segmentation, and quantification of the most remarkable stroke consequences (definition of lesion area and edema), but only iodine-stained brain images could be used for automatic segmentation of white matter fiber tracts and infarct lesions. Alongside their effectiveness, a contrast agent should be chosen according to safety and handling^37^; stains based on inorganic iodine are easier to handle and less toxic than osmium tetroxide, penetrate faster into the tissue and produce higher contrast in x-ray images, allowing manual/automatic segmentation.

Micro-CT imaging, combined with the contrasting agents, achieves data similar to MRI, which is a long, expensive and intensive process. Micro-CT also delivers higher voxel diameter resolution and guarantees that the cerebral morphology remains intact for the construction of 3D models, with a high level of detail and resolution^58^. Comparing micro-CT and micro-MRI ex-vivo scans reported in the literature, the micro-CT scan of a brain takes around 5 h to complete, whereas micro-MRI takes up to 12 h, at an isotropic resolution of 62.5 µm^59^. Additionally, MRI takes from 24 h to 2 weeks for sample processing, while micro-CT takes 3 to 5 h to provide a complete visualization of the brain’s external and internal morphology. However, micro-CT, at this level of resolution, can only be performed ex-vivo and post-mortem, whereas MRI permits longitudinal assessment of the lesion in the same individual.

Micro-CT also offers important advantages compared to histological procedures to evaluate ischemic lesions: processes a large number of brains- only requires that samples are exposed to different solutions; acquires images with optical slices of 3.5–4 µm-thinner than physical histological slices of 0.4–1 mm; acquires high-resolution images with 3D information compared to 2D for histological procedures; discriminates the core and penumbra areas, and delineates penumbra border to healthy tissue^60^. Histological methods for infarct analysis usually comprise brain slicing followed by either enzymatic labeling of live cells (as in TTC, using fresh tissue) or immunolabeling for specific cellular markers (as NeuN or MAP2 for neurons, using fixed tissue), producing images with high-resolution to the cellular and subcellular level, but with tissues suffering shrinkage and deformation due to processing, besides the loss of information due to tissue slicing^1^. In iodine-stained brains, shrinkage is also a drawback, although it retains brain 3D morphological information, and may allow further processing using histological procedures, such as immunolabelling (supplem. Figure 3B—some optimization of the dehydration/rehydration process is required), thus extending the 3D virtual histological results by precise identification and correlation of different cell types to anatomical structures visualized in the micro-CT^37,45,61^. We validated the quantification of brain lesions by micro-CT using osmium staining by a positive correlation with the widely used method of TTC histology, indicating that the total ischemic lesion identified is similar.

The TIA model is defined by the successful occlusion of the MCA without neurologic deficit or lesion, 24 h after reperfusion. This model was not broadly studied, since no morphological alterations are observed using TTC staining or MRI, and only cellular/in-situ methods (such as Haematoxylin Eosin stain, and TUNNEL) can identify ischemic alterations. TIA causes functional deficits in human patients, and the majority of the studies using the TIA mice model agree that short-term ischemia leads to selective neuronal loss in the striatum^7,34^. White matter injury, characterized by demyelination and axonal breakdown, was also found after MCAO in rats, and this injury played a critical role in these rodents poststroke disability^12^. Segmentation of lesions constitutes a prerequisite for quantitative analysis in stroke research, a tedious, time-consuming (limiting sample size), and bias/error-prone task when performed manually. The present work addresses the need for reliable, semi-automatic, or automatic methods to successfully segment and quantify stroke lesions^62^. We describe a novel method to quantify (semi-automatic protocol) white matter degeneration in the striatum after 10-min of middle cerebral artery occlusion in mice, by determining white matter volume by considering the caudate putamen volume and detecting a decrease in the white matter volume in the affected hemisphere. As manual input in the first phase of the segmentation procedure is still required to segment the striatum in each brain hemisphere, a human-originated error can be introduced into the evaluation. To be objective, the manual segmentation was performed with the Allen Mouse Brain Atlas as a reference. In the following step, we apply thresholding in the both brain hemispheres simultaneously; we can expect the quantitative evaluation of the white matter volume fraction to be consistent between brain hemispheres.

The deep learning-based segmentation of the lesion core and penumbra allows a fully automatic and consistent evaluation of the lesion volume across multiple samples, avoiding the time-consuming manual segmentation process. We show that ground image segmentation masks are not necessary to train and apply to the lesion segmentation model; by simulating the lesion on the healthy brain hemisphere, we obtain volume measurements that correlate with those obtained by manual segmentation.. The limitations of the method relate to operator-dependent separation of the healthy brain hemisphere from the hemisphere affected by stroke, and segmentation performed on images, which were down-sampled 10 times to reduce computer requirements, without data prejudice (considering high-frequency images, as obtained in micro-CT, are not required for lesion detection).

Lesion size and location affect the magnitude of impairment and recovery following stroke^6^. Co-registration of the volumetric micro-CT images with Allen Mouse Brain Atlas for the identification of neuroanatomical brain structures, together with behavioral functional assessments, contributes to increased translation into clinical practice. Previously we described functional tests to evaluate sensorimotor and cognitive disabilities in stroke pre-clinical models^63^. This co-registration is still manual and should be considered with caution, due to brain edema on the ipsilateral side. Nonetheless, it allows the identification of major sub-anatomical brain areas affected in the tMCAO stroke model, including the striatum and the somatosensory cortex, among others (Fig. 8). Interestingly, affected areas can be correlated with the functional deficits mice presented, and compared to human patients^18,56^. Registrations of ischemic lesions in stroke models to brain atlas were only previously performed on MRI images^18^, and as mentioned, MRI images are prone to distortion. Before our study, no such registration was performed using more accurate micro-CT images.

In conclusion, we describe micro-CT as a powerful tool for easy, fast, 3D imaging of the whole mouse brain, with high-resolution and detail. Iodine and osmium stain of mouse brains subjected to different ischemia models allowed the characterization of ischemic lesion evolution over time. We also describe a manual quantification of the ischemic lesion, with clear discrimination of the core and penumbra areas, and a positive correlation with the quantification by TTC staining. Iodine contrast allowed for the development of an automatic quantification protocol of core/penumbra volumes in stroke brains, as well as the characterization of striatum white matter degeneration in TIA brains. Therefore, the novel methodology characterized in the present work constitutes a major asset to preclinical stroke studies.

## Material and methods

### Mice

The number of mice handled for the presented work was approved by the Institutional and National General Veterinary Board (DGAV) and i3S internal Ethical Committee (approval reference number 003424), according to National and European Union rules. Three- to 12-month-old, male/female wild type C57BL6 mice were used. The animals were reproduced, maintained (regular rodents chow and tap water ad libitum) and experimentally manipulated under a 12 h light/dark cycle in type II cages in specific pathogen-free conditions in the animal facility (microbiological health status available). The method of euthanasia used was lethal anesthesia with 170 mg/kg ketamine/2 mg/kg medetomidine via (ketamine + medetomidine) intraperitoneal administration. Charles River Laboratories is the external animal facility used to acquire animals. Physical randomization for group selection was performed using a bioinformatics tool available from Graphpad Prism^®^ QuickCalcs website: https://www.graphpad.com/quickcalcs/randomize1/. The order by which the animals from the different experimental groups were assessed was random during each experimental session. ARRIVE guidelines were taken into consideration in experimental reporting. This study was not pre-registered.

### tMCAO

Mice were anesthetized with isoflurane (4% for induction and 2–1.25% for maintenance) in a mixture of N_2_O:O_2_ (70:30) using a small-anesthesia system. Rectal temperature was maintained at 37.2 °C throughout the surgical procedure making use of a rodent warmer with a rectal probe (STOELTING, Wood Dale, IL, USA).

Cerebral blood flow (CBF) was monitored trans cranially using a laser Doppler flowmeter (LDF, PeriFlux System, PERIMED, Stockholm, Sweden). Animals were placed under a stereo microscope (Leica S8 APO, Leica Microsystems, Wetzlar, Germany) and fur from the mice’s nape was shaved and disinfected. An incision of 1–2 cm was made on the clean skin, and the margins of the incision were pulled laterally to reveal the cranium. A 0.5 mm diameter microfiber Laser-Doppler probe (Master Probe 418-1connected to microtip: MT B500-0L240) was attached to the skull with cyanoacrylate glue 6 mm lateral and 2 mm posterior to bregma. While under general anesthesia, regional cerebral blood flow was monitored within the MCA territory. The surgical procedure was considered adequate if ≥ 70% reduction in blood flow occurred immediately after placement of the intraluminal occluding suture and reperfusion occurred after the occlusion period; otherwise, mice were excluded.

Transient middle cerebral artery occlusion was induced by the intraluminal suture method^51,64–66^. Briefly, after a laser-Doppler probe was attached to the skull, animals were placed in a supine position, under the stereo microscope. The surgical region of the neck was shaved and disinfected. A midline neck incision was made, followed by dissection of the underlying nerves and fascia to expose the right common carotid artery (CCA). The CCA was carefully separated from the vagus nerve, which lies laterally to it, taking extra care not to damage/stimulate or puncture it with the surgical tools. The right external carotid artery (ECA) and internal carotid artery (ICA) were also isolated by blunt dissection of the surrounding tissues (avoiding the rupture of the superior thyroid and occipital arteries). Afterward, the CCA was temporarily ligated with a silk suture and the ECA was permanently ligated as distal as possible from the bifurcation of the CCA, and loosely ligated proximal to the bifurcation. A reduction in cerebral blood flow was observed. A microvascular clamp was applied to the ICA. A small incision was made in the ECA between the silk sutures, and a new silicone-rubber coated 6–0 nylon monofilament (^21–23^ 6023PK10 DOCCOL Corporation, Massachusetts, USA) was introduced into the ECA and pushed up the ICA until the filament was ≈9 mm from the place of insertion (marked with a silver sharpie) and a sudden drop was observed in the blood flow, effectively reaching the circle of Willis and blocking the MCA. The coating length of the monofilament lay between 2–3 mm, and the tip diameter depended on the animal’s weight. The loose suture around the ECA was then tightened around the inserted filament to prevent movement during the occlusion period, which lasted for either 45 or 10 min of occlusion. After the designated occlusion time was over, the filament was removed and the proximal section of the ECA was permanently tied. The temporary tie around the CCA (only closed when necessary to avoid blood leakage through the ECA hole) was removed and reperfusion was established. This was observed in the Laser-Doppler as a rise in the regional CBF. Mice were administered 0.2 mL of bupivacaine (2 mg/kg in 0.9% NaCl) in the neck open wound. The Laser-Doppler probe was cut close to the skull and both the neck and head incisions were sutured. As a control, sham-operated animals were subjected to the same procedure without occlusion of MCA.

Immediately after surgery, 0.5 ml saline (0.9% NaCl) and buprenorphine (50 µL/10 g of animal weight—0.08 mg/kg) was given subcutaneously to each mouse as a fluid replacement and as a form of analgesia, respectively. Buprenorphine was repeatedly administered at time intervals of 8 to 12 h for the first 3 days post-ischemia; two daily administrations of 0.3 mL of both 5% glucose in 0.9% NaCl and 50/50% Duphalyte in NaCl were given until mice recovered weight. Right after surgery, mice were placed in a recovery box (Small Animal Recovery Chamber/Warming Cabinet, HARVARD APPARATUS) maintained at a humidified temperature of 36.8 °C until fully recovered from anesthesia (~ 2 h). To reduce stress, some elements such as paper from the home nest and roll papers were placed in the recovery box. When anesthesia completely wore off, recovery box temperature was decreased to 34.8° for the next 12 h; afterward, home cages were half placed on a heating pad for the first day post-surgery, allowing mice to choose their environment during recovery, regulating body temperature and controlling post-stroke hypo/hyperthermia. When mice were returned to their home cage, significant care was taken into pairing mice with their pre-surgery cage mates. However, we separated sham-operated animals from tMCAO-operated animals to avoid stress and fighting within the cage, due to differences in the alertness and motile/cognitive deficits between the animals. The mortality rate was 10% (in tMCAO 45 min only). After 3-, 24- or 72 h of recovery, animals were euthanized.

### Osmium tetroxide stain

In the Osmium tetroxide cohort, mice were deeply anesthetized and were perfused transcardially (using 20 ml syringes, through high-pressure manual delivery, keeping heart inflated) with 40 mL PBS, followed by 40 mL of 2% PFA + 2.5% glutaraldehyde with 4% sucrose in PBS, pH 7.4. The whole brain was removed and fixed in 30 mL of the same fixation solution for at least 1 week, at 4 °C, in a 50 ml conical centrifuge tube.

After the post-fixation period, brains were washed in water to remove fixatives and PBS, and stained in an aqueous 2% osmium tetroxide (O_s_O_4_) solution (15 ml for each mouse brain) in a 50 ml conical centrifuge tube for two weeks, at 4 °C in a horizontal shaker. Since osmium tetroxide is toxic if inhaled, tubes were sealed with Parafilm^®^ to minimize evaporation, and osmium tetroxide manipulation was always performed in a fume hood. When performing the staining, tubes were protected from direct light with aluminum foil. After staining, brains were washed, wrapped in Parafilm^®^ (to prevent dehydration during micro-CT scanning), and transferred to Parafilm sealed 5 ml tubes (SARSTEDT, 62.558.201) for µCT scanning, for short-term storage. If long-term storage is needed, brain dehydration followed by resin infiltration and embedding should be performed, as described by Masís et al^67^. In this work, short-term storing was the chosen option, mainly because one of the parameters to be analyzed was the brain edema after tMCAO. As such, the dehydration procedure could influence the measurement of this parameter, even though edema is always calculated in comparison with the contralateral hemisphere of the corresponding brain.

Detailed information regarding all the steps involved in *ex-vivo* mouse osmium tetroxide staining is available in Table 4.

**Table 4:** **Detailed protocol for whole mice brain preparation for microCT with osmium tetroxide.**

Step	Solution	Time (min)	Temp. (°C)
**Perfusion**			
Lethal anesthesia	Ketamine/medetomidine, IP	5	22
Blood perfusion	PBS	10	22
Brain fixation	2% PFA, 2.5% GA, 4% sucrose, in PBS	10	22
**Post-fixation**			
Fixation	2% PFA, 2.5% GA, 4% sucrose, in PBS	7–30 days	4
Washing	Distilled H_2_O	1, 1, 1, 15	22
**Staining**			
Osmium	2% OsO_4_, in H_2_O (15 mL/brain)	15 days	4
**Micro-CT prep**			
Washing	Distilled H_2_O	1, 1, 1, 15, 15, 15	22
Preparation	Parafilm^®^ wrapping, and 5 mL_Tube accommodation	5	22
**Brain Storing**			
Short-term	Protected from light; Sealed container	3–12 months	4
Long-term	Dehydration; Resin embedding	Years	22

### Micro-CT scanning for osmium stained brains

All osmium tetroxide stained ex-vivo mouse brains were scanned using a BRUKER micro-CT scanner (SkyScan1276, BRUKER, Belgium), from the i3S Scientific Platform Bioimaging. The settings used were: voltage of 90 kV and an output current of 47 µA; 4 µm spatial resolution, 0.2° rotation step through 360 degrees, giving rise to 1801 projections; an Aluminum filter of 1 mm was used, together with a frame averaging of 4 The scanning of each brain took around 5 h.

Reconstruction of scanned brain images was performed using the NRecon software (version 1.7.4.2, BRUKER, Kontich, Belgium). The settings were normalized for all the brains, however fine tuning was performed for each brain to improve the quality of deconvolution not achieved with normalized automatic deconvolution. Fine-tuning parameters were: Smoothing of 0; Misalignment compensation between 52 and 60; Ring artifacts reduction between 4 and 6; and Beam-hardening correction between 20 and 30%.

### Iodine staining

After the aforementioned recovery times, the animals from the iodine cohort were anesthetized and transcardially perfused (using 20 mL syringes, through manual delivery – keeping heart inflated) with 40 mL PBS, followed by 20 mL of 4% PFA in PBS, pH 7.4. Brains were carefully dissected and post-fixed in 20 mL of the same fixation solution for at least 3 days, at 4 °C, in a 50 ml conical centrifuge tube.

Brains were dehydrated in ethanol grade series (30%, 50%, 70%, 80%, 90%) for 2 h followed by 24-h staining in 90% methanol plus 1% iodine solution, in agitation, at room temperature. Since iodine solutions are not toxic, no protective measurements were not needed.

After staining, brains were quickly rehydrated with 30% and 70% ethanol, for 1 h each, at room temperature with agitation. Finally, they were wrapped in Parafilm^®^ (to prevent further dehydration during the micro-CT scan) for micro-CT scanning. For short-term storage, brains were kept in 5 mL tubes (Sarstedt, 62.558.201) at 4 °C. If long-term storage is needed, brain dehydration, followed by resin infiltration and embedding should be performed, as described by Masís et al^15^. In this work, short-term storage was the chosen option.

Detailed information regarding all the steps involved in *ex-vivo* mouse iodine stain is available in Table 5.

**Table 5:** **Detailed protocol for whole mice brain preparation for micro-CT with iodine.**

Step	Solution	Time (min)	Temp. (°C)
**Perfusion**			
Lethal anesthesia	Ketamine/medetomidine, IP	5	22
Blood perfusion	PBS	10	22
Brain fixation	4% PFA, in PBS	10	22
**Post-fixation**			
Fixation	4% PFA, in PBS	7-60 days	4
**Staining**			
Dehydration	30%, 50%, 70%, 80%, 90% ethanol	2 h, each	22
Iodine	1% iodine, in 90% metanol (15 mL/brain)	24 h	22
**micro-CT prep**			
Preparation	Parafilm^®^ wrapping	2	22
**Brain Storing**			
Short-term	Protected from light; Sealed container	3–12 months	4
Long-term	Dehydration; Resin embedding	Years	22

### Micro-CT scanning for iodine stain

Iodine stained *ex-vivo* mouse brains were scanned using the BRUKER micro-CT scanner (SkyScan1276, BRUKER, Kontich, Belgium), from the i3S Scientific Platform Bioimaging, where the settings used were: a voltage of 85 kV and an output current of 200 µA; 3 µm spatial resolution, 0.2° rotation step through 360°, giving rise to 1801 projections; an Aluminum filter of 1 mm was used, together with a frame averaging of 8. Scanning of each brain took around 3 h.

Reconstruction of scanned brain images was performed using the NRecon software (version 1.7.4.2, BRUKER, Kontich, Belgium). The settings were normalized for all the brains, however fine-tuning was performed for each brain to improve the quality of reconstruction not achieved with normalized automatic deconvolution. Fine-tuning parameters: Smoothing of 0; Misalignment compensation between 52 and 60; Ring artifacts reduction between 4 and 6; and Beam-hardening correction between 20 and 30%.

For higher resolution scans, used for striatum fiber automatic segmentation, stained samples were fixed in 1% agarose gel (TOP-BIO s.r.o., Prague, Czech Republic) and placed in 1.5 mL centrifuge tube. GE Phoenix v|tome|x L 240 (WAYGATE Technologies GmbH, Huerth, Germany) was used for the acquisition. The micro-CT scan was carried out in an air-conditioned cabinet (21 °C) at 60 kV acceleration voltage and 200 μA tube current. Exposure time was 700 ms and 3 X-ray projections were acquired in each angle increment and averaged for reduction of noise. The achieved resolution was 4.5 μm with isotropic voxels. Tomographic reconstruction was performed by using the software GE phoenix datos|× 2.0 (WAYGATE Technologies GmbH, Huerth, Germany). The data was then imported to VG Studio MAX 3.4 (VOLUME GRAPHICS GmbH, Heidelberg, Germany)—https://www.volumegraphics.com/en/products/vgsm/what-s-new-in-vgstudio-max-3-4-x.html.

### Manual segmentation and quantification of total lesion/core volume and edema using the CTAn software

Fine adjustment of the 3D volume images was performed using the DataViewer software (version 1.5.6.2, BRUKER, Kontich, Belgium), followed by volumetric analysis was performed on CTAn software (version 1.20.3.0, BRUKER, Kontich, Belgium). For edema extent calculation, brain hemispheres were manually annotated in the transaxial orientation every 10 slices. The software rendered the region of interest in every slice through interpolation and calculated object volume. In tMCAO 45-min brains, total lesion and core, when present, were manually delineated in the transaxial orientation of every 10 slices. Once again, the software adjusted the region of interest in every slice through interpolation and calculated object volume. Edema extent was calculated by applying the equation: Edema extent (% of the contralateral hemisphere) = ((V_ipsilateral_ (µm^3^)−V _contralateral_ (µm^3^))/V_contralateral_ (µm^3^))*100; and lesion volume was corrected for edema by applying the equation: Total lesion (% of brain volume) = (V_lesion_ (µm^3^) x (V_Contralateral_ (µm^3^)/V_Ipsilateral_ (µm^3^)*100)/V_brain_ (µm^3^); with V being the volume obtained during image quantification and V _brain_ as the sum of V_contralateral_ and V_ipsilateral_, based on^15^.

### Semi-automatic segmentation and quantification of white matter degeneration in the striatum

The first step required for the analysis of the white matter fibers was their segmentation in the whole CT volume. This segmentation was performed using the software Avizo 2020.2 (THERMO FISHER SCIENTIFIC, Waltham, MA, USA). The tomographic slices were pre-processed by utilizing the non-local means algorithm for the reduction of noise. Then the caudate putamen was manually segmented in coronal tomographic slices utilizing the coronal Allen Mouse Brain Atlas as reference^68^. Every 10th slice was manually annotated, and the in-between slices were computed by linear interpolation. These manually segmented volumes were used as a region of interest for subsequent segmentation of the white matter fibers. A semi-automatic approach was used for the segmentation of the whiter matter: white top-hat transform. White top-hat transform detects light areas in the image using morphological operators. We utilized a ball-shaped structuring element 19 voxels in diameter. The top-hat-transformed image was then manually thresholded. This segmentation was performed simultaneously for both hemispheres, which allows accurate comparison. For the semi-quantification, a simple volume fraction analysis was performed in the Avizo 2020.2 (THERMO FISHER SCIENTIFIC, Waltham, MA, USA), where the volume of the white matter was measured in relation to the volume of the manually segmented caudate putamen. The segmented striatum masks were then imported to VG Studio MAX 3.4 (VOLUME GRAPHICS GmbH, Heidelberg, Germany) and wall thickness analysis was performed. For each voxel, the largest sphere inscribed to the white matter volume was determined, as containing the center position of the evaluated voxel. See Fig. 6A for an overview of the proposed workflow.

### Automatic segmentation of the total lesion and core

An unsupervised deep learning-based approach for the segmentation of the total lesion and core volume was utilized for the automatic evaluation (See Fig. 6B). For the method to be applied, two conditions must be met: (1) the lesion should be isolated in one brain hemisphere, (2) the user must be able to distinguish the affected hemisphere from the healthy one. First, the brain midline is manually detected, and the brain scans are separated into two subvolumes along this midline. The subvolumes are all cropped to be of a unified size of 1920 × 1280 pixels and downsampled 10 times to lower the computational requirements. The hemispheres unaffected by stroke form the training database for the deep learning model. We use a U-net-shaped CNN^69^ (See Fig. 6C for the schematic representation of the CNN utilized in our work). The size of the CNN input and output is 192 × 128 pixels. The same as in the original U-net implementation, the CNN consists of an encoding and a decoding part. We use 3 × 3 convolutional kernels followed by 3 × 3 strided convolutions (stride 2) in the encoder to perform the repeated down-sampling of the extracted feature maps instead of the max pooling performed in the original implementation. In the decoding part, we apply 3 × 3 convolutional kernels and the up-sampling is performed by 3 × 3 transpose convolution layers with stride 2. The number of convolutional kernels in the first layer of the CNN is 64 and increases up to 1024 in the deepest part of the network. The feature maps from the encoder are concatenated with the decoder feature maps to preserve the spatial information from the input images during the training and inference. ReLU activation function is applied after each convolution. The input to the neural network is a coronal 2-D micro-CT cross-section of the brain hemisphere, where a lesion-like area is randomly simulated by subtracting two randomly generated concentric discs deformed by the elastic transform proposed in^70^ (parameter alpha is set to 120 and sigma to 5) and blurred with by gaussian blurring with random parameter sigma (range from 5 to 20). The outermost ring simulates the whole lesion and the inner ring stimulates the lesion core. The radius of the outer circle is defined randomly in the range from 20 to 80 pixels and its center is randomly set anywhere within the image. The radius of the inner circle is also defined randomly in the range from 0 to 50% of the outer circle radius. The decrease in intensity in the simulated lesion is random from 0 to 50% of the original intensity and is decreased further by up to 50% in the simulated lesion core.

The target of the CNN training is the original CT image of the healthy hemisphere without the simulated lesion area. The CNN thus learns to transform the simulated lesioned brain image into an approximation of images of a healthy brain. The CNN is trained on a total of 830 micro-CT images with randomly simulated lesions for 100 epochs with batch size 32 using the mean-squared-error loss and Adam optimizer^71^ with an initial learning rate of 0.001 (see Supplemental Fig. 6A). After the network is trained, it can be applied to real lesioned hemisphere image data. The CNN transforms the real 2D micro-CT cross-section of the stroke-affected brain hemisphere into an approximation of a healthy brain hemisphere. By subtracting the lesioned image from the output of the CNN, a lesion probability map is obtained. This probability map is then automatically thresholded in whole 3D volume by Li thresholding^72^ to obtain the whole lesion area in 3D (see Supplemental Fig. 6B for visualization of the segmentation in selected tomographic cross-sections). The threshold for the lesion core is obtained as:

$$T_{{\text{core }}} = \frac{3}{2}T_{{{\text{lesion}}}}$$

The largest connected component is selected for both the whole lesion area and lesion core thresholded image. The 3D segmented volumes are finally upsampled 10 times by bi-cubic interpolation and form the final segmentation masks and total lesion and core volume can then be computed. Nvidia Quadro P5000 with 16 GB of graphical memory was utilized for training the CNN on a system equipped with 512 GB of RAM and Intel^®^ Xeon^®^ Gold 6248R CPU. The proposed CNN was implemented in the programming language Python (version 3.7.9) using the library Keras^73^ (version 2.3.1) and Tensorflow backend^74^ (version 2.1.0). CUDA (version 10.1) and CUDnn (version 7.6.5) were used for GPU acceleration of the training and inference process. NumPy^75^, scikit-image^76^ and Pillow libraries were used for manipulating and transforming the image data.

### TTC stain

In the TTC cohort, infarct volume was evaluated as previously described in^55^ with minor changes. After the mice were deeply anesthetized, the brain was removed, and the forebrain sliced into 1-mm-thick sections using a mouse brain slicer (Acrylic brain Matrix, Coronal, 40–75 g, STOELTING, USA) on ice. The sections were rinsed once in ice-cold 0.9% sodium chloride (NaCl) for 10 min and subsequently immersed in 25 mL of 0.01% 2,3,5-triphenyl tetrazolium chloride (TTC) (Sigma-Aldrich, USA) in 0.9% NaCl at 37 °C for 15 min. Slice images were acquired with a camera and analyzed *à posteriori*. The unstained area in the section was regarded as an infarct area whereas the stained area was considered a non-infarct area.

Infarct volume was determined using a semi-automated quantification method in Fiji-ImageJ^77^. First, images were calibrated using a millimetric piece of paper as a reference, captured in all photos next to the slices. Next, images were batch processed and analyzed in a custom-made macro. Each image containing several slices of the same condition was pre-processed to isolate each slice, consisting of the manual selection of a color threshold, followed by a Gaussian filter (sigma 10), thresholded with Huang’s method, and the morphometric operations fill holes and dilation. The output of the preprocessing step is the centroid of each slice. Finally, each slice was segmented alone by duplicating a rectangle, centered in the detected centroids, with size *w x h* (*w* = original image width, *h* = original image height/number of slices).

The infarct lesion measurement of each slice was performed by a blind experimenter with the following steps: (i) manual delineation of the middle line to divide the slice into two hemispheres; (ii) manual annotation of the stroke area (when present); (iii) quality control of the resulting regions of interest. The macro output obtained was a results table with slice labels, the contra- and ipsilateral hemispheres area, and the infarct lesion area. Volumes were obtained by summing the area of the infarct brain segment from all brain slices (considering that each slice’s thickness is 1 mm). Edema extent and corrected lesion volume were calculated as described above and based on^78^.

### Image registration to allen mouse brain atlas

The whole image series from a tMCAO mice brain (stained with osmium) were registered to match the cutting plan and proportions of the atlas sections using the open-source serial aligner software QuickNII^79^ (https://www.nitrc.org/projects/quicknii/). This software allows the assignment of spatial location (features such as angle, and size can be adjusted) so that atlas can match with section images. After, the whole image series segmented with the atlas was saved, and structures affected by an ischemic lesion in this mouse were identified.

### Brain slicing and immunolabeling

Brains were post-fixed in the same fixation solution, overnight, at 4 °C and then left in 30% sucrose in PBS at 4 °C, until they sunk in the solution. Coronal Sections (40 µm) were cut on a freezing microtome (LEICA Cryostat CM 3050) and used for free-floating immunohistochemistry. Slices were incubated with 10% horse serum, and 0.2% Triton X-100 in PBS, for permeabilization and blocking, for 1 h, in agitation, at room temperature. Primary antibodies were always incubated for 48–72 h at 4 °C, in PBS. Secondary antibodies were incubated overnight at 4 °C. The slides were mounted on gelatin-covered glass slides with a fluorescent mounting medium (DAKO) and imaging was performed on a laser scanning Confocal Microscope Leica SP8 (LEICA microsystems, Wetzlar, Germany), using the × 10 air objective. In each set of experiments, the same batch of antibodies (primary and secondary) were used, and images were taken using the same settings. Primary antibodies used were anti-NeuN (1:500, Mouse, CHEMICON international, ab377), anti-MAP2 (1:500, Rabbit, ABCAM, ab246640), and anti-BrdU (TUNEL Assay Kit, ABCAM, ab66110); as secondary antibodies, Alexa Fluor 488 and 568 (1:500) were used. The fluorescent dye Hoechst 33,342 (0.5 μg/mL–15 min room temperature) was used to stain nuclei.

Brains stained with iodine were rehydrated in two, 2-h, PBS steps and then left in 25% sucrose in PBS at 4 °C overnight. Coronal sections were cut and immunolabeled as described above.

### Statistical analysis

Statistical analysis was performed using GraphPad Prism Version 8.0/9.0 (GRAPHPAD Software, Inc., San Diego, CA, USA). Data were collected from experiments and presented as mean ± SEM with individual points, “n” information (the experimental unit) is described in figure legends. The experimental unit was each single animal. Linear regression analysis was performed to correlate edema extent and total lesion values obtained either through microCT or TTC. A previous power analysis was performed to obtain at least 25% difference (10% SD) when comparing two groups, with 90–95% power. This analysis pointed to experimental groups including 3 and 6 animals. Statistical analysis was performed using one-way ANOVA, followed by Dunnet’s or Sidak’s multiple comparisons tests: ****p < 0.0001, **p < 0.01. Identification and removal of outliers were performed using automatic GraphPad prism software 8.0, using the ROUT (robust nonlinear regression) method, with a Q = 1% (recommended).

## Supplementary Information

Supplementary Information 1.

Supplementary Information 2.

Supplementary Video 1.

Supplementary Video 2.

Supplementary Video 3.

## Data Availability

The data supporting the findings of this study are available within the article, as supplementary data or can be available, upon request (due to high Gigabytes of data) from the corresponding author João Gomes.

## Abbreviations

tMCAO: Transient Middle Cerebral Artery Occlusion
TIA: Transient ischemic attack
MRI: Magnetic resonance imaging
micro-CT: Microcomputed tomography
PMA: Phosphomolybdic acid
PTA: Phosphotungstic acid
MCA: Middle cerebral artery
CCA: Common carotid artery
ECA: External carotid artery
ICA: Internal carotid artery
TTC: 2,3,5-Triphenyl tetrazolium chloride
