# Supplementary material for: High-resolution micro-CT for 3D infarct characterization and segmentation in mice stroke models
Source: Sci Rep. 2022 Oct 19;12:17471. doi: 10.1038/s41598-022-21494-9 (PMC9582034; doi:10.1038/s41598-022-21494-9)

Suppl. Fig. 1- High-resolution micro-CT imaging of mice brain subjected to mouse stroke model (tMCAO) using iodine staining.

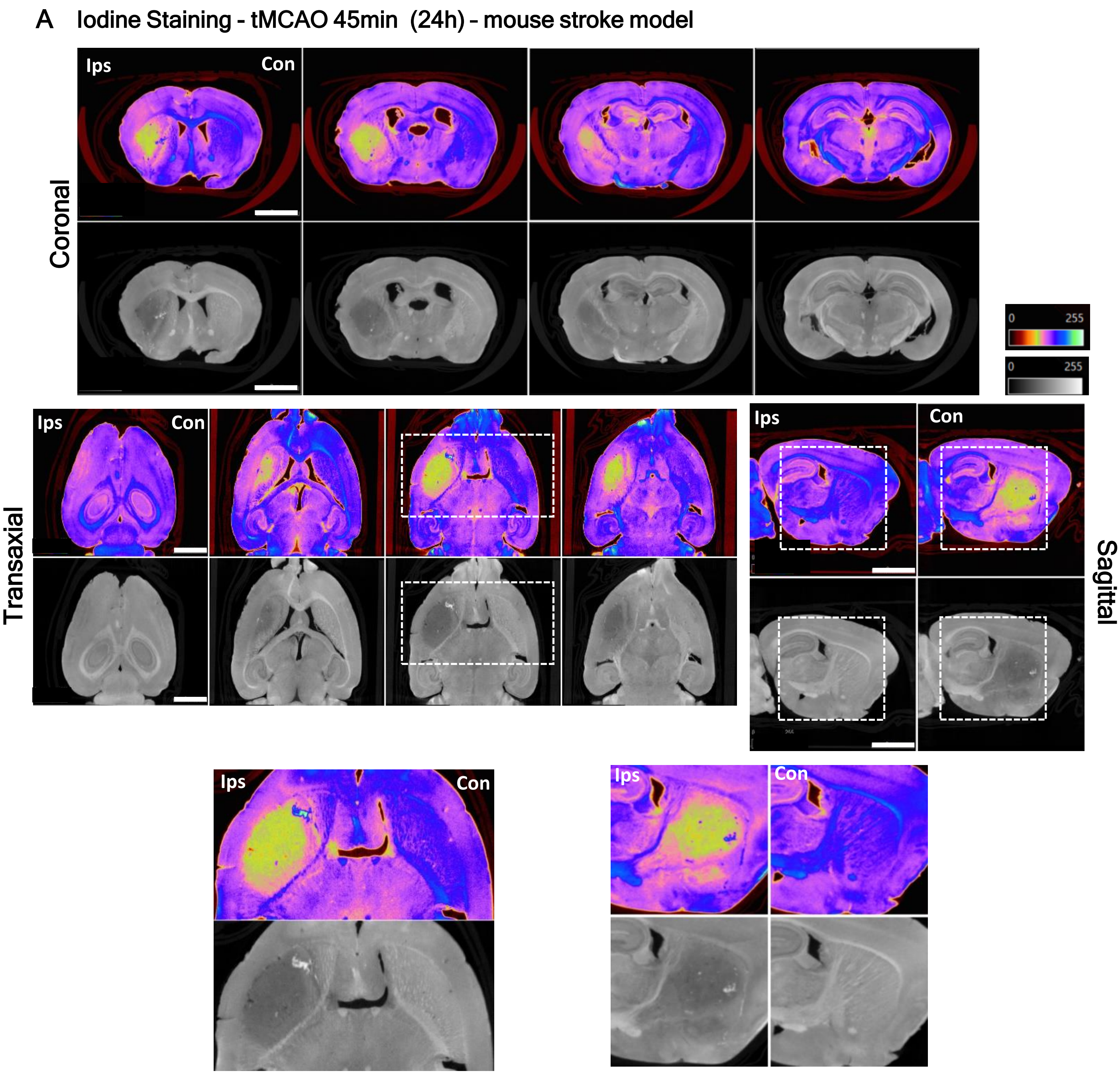

Suppl. Fig. 2- Iohexol, phosphotungstic acid (PTA) and phosphomolybdic acid (PMA) staining of whole mice brain.

A Iohexol staining in tMCAO 45’ (24 h) mice

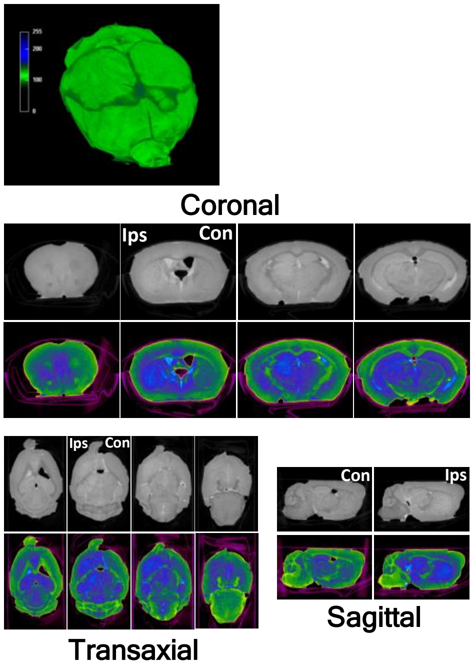

B Phosphotungstic acid (PTA) staining in tMCAO 45’ (24 h) mice

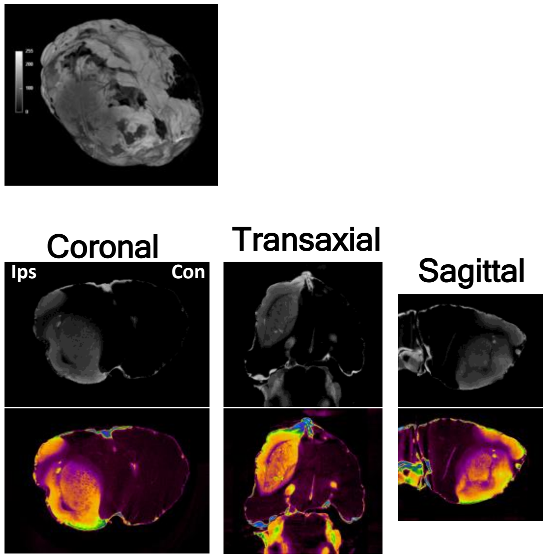

C Phosphomolybdic acid (PMA) in PBS staining in naive (no-surgery) mice

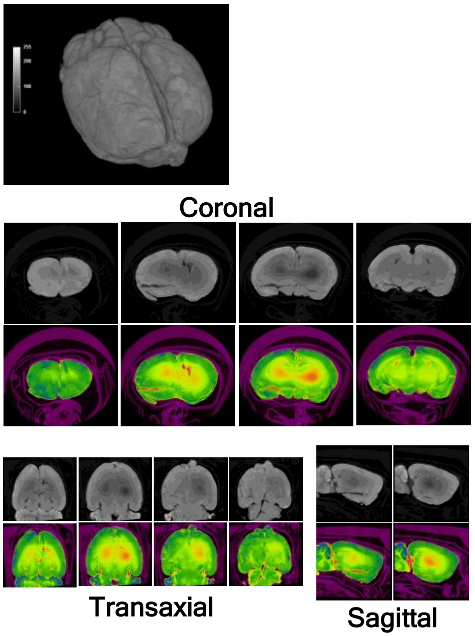

Suppl. Fig. 3- Sequential immunostaining process in iodine-stained mice brains

A

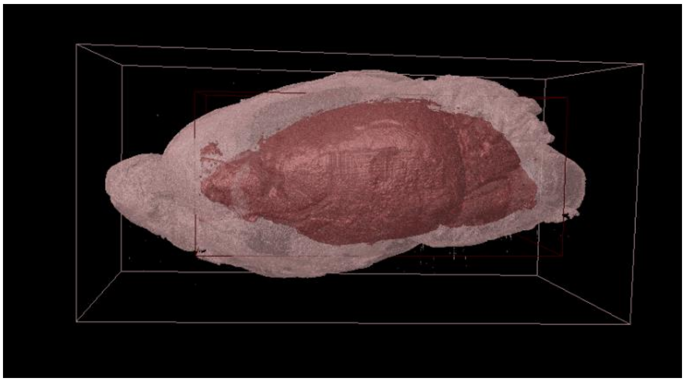

B

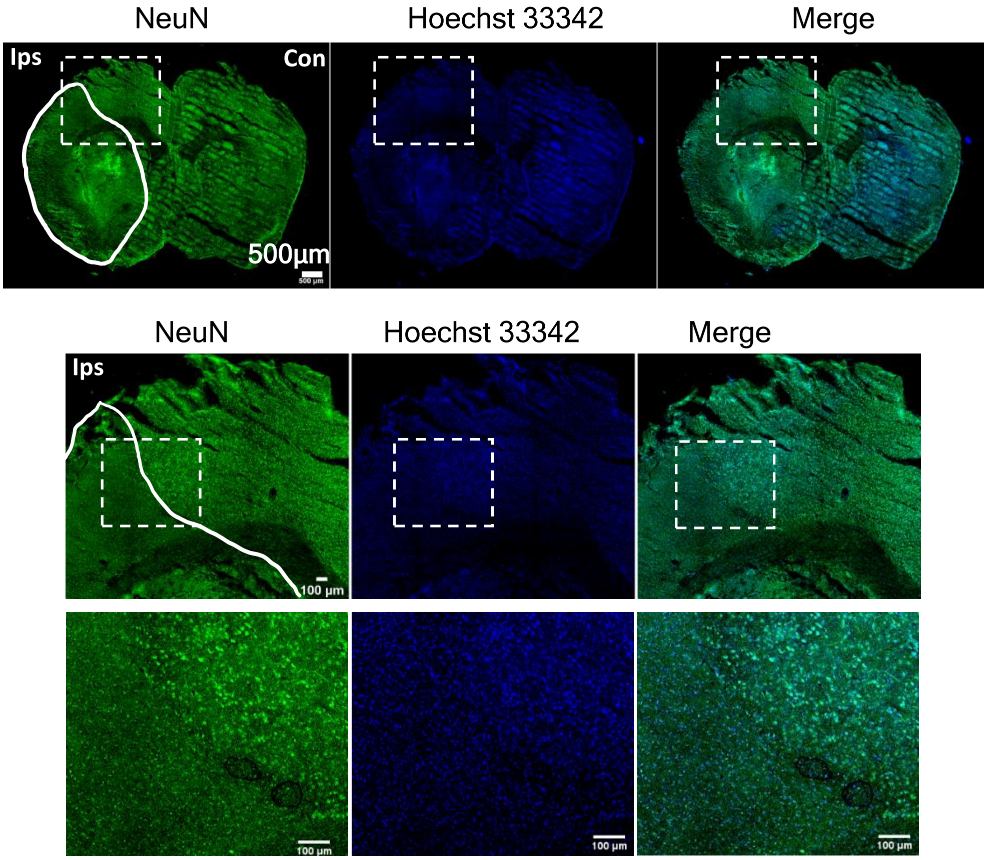

Suppl. Fig. 4- High-resolution micro-CT imaging of mice brains subjected to the TIA mouse model using Osmium Tetroxide staining.

A Osmium Tetroxide Staining - TIA mouse model

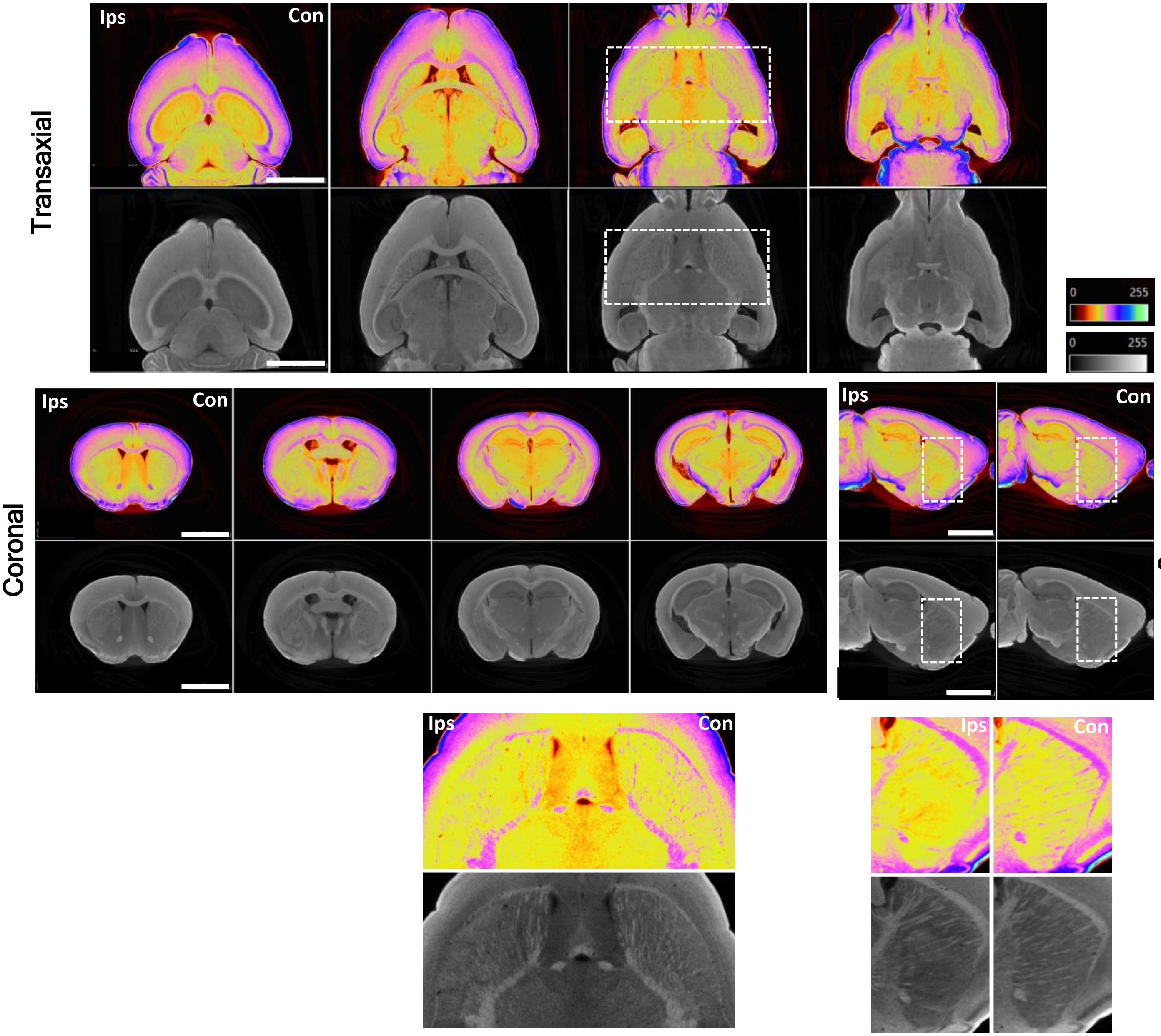

Supplementary Fig. 5- Brain ischemic lesions (stroke and TIA models) progression using high-resolution micro-CT.

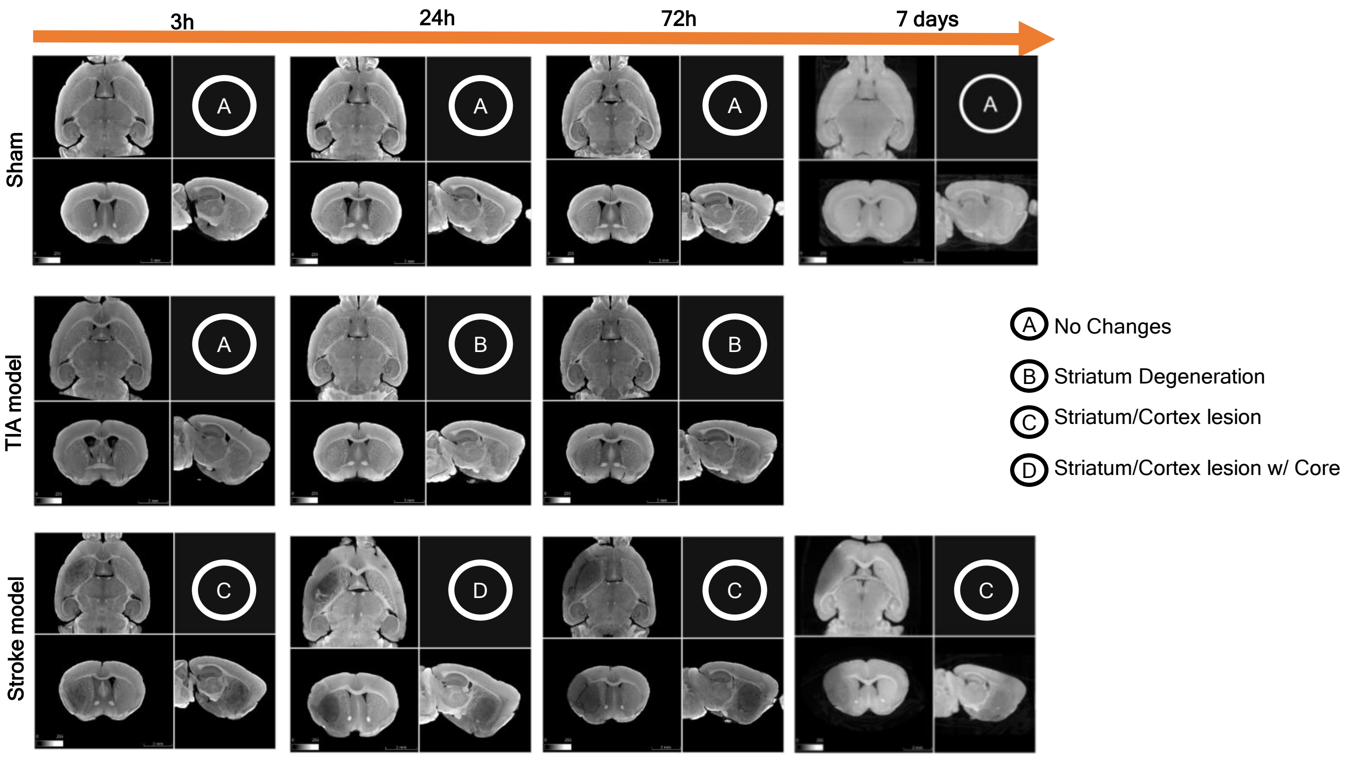

Suppl. Fig. 6 – Neural network training and results visualization

A

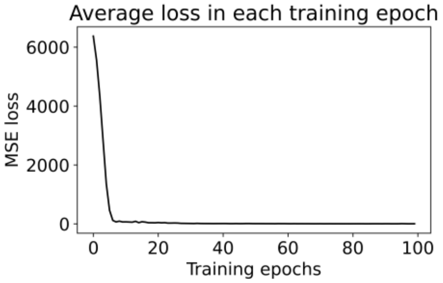

B

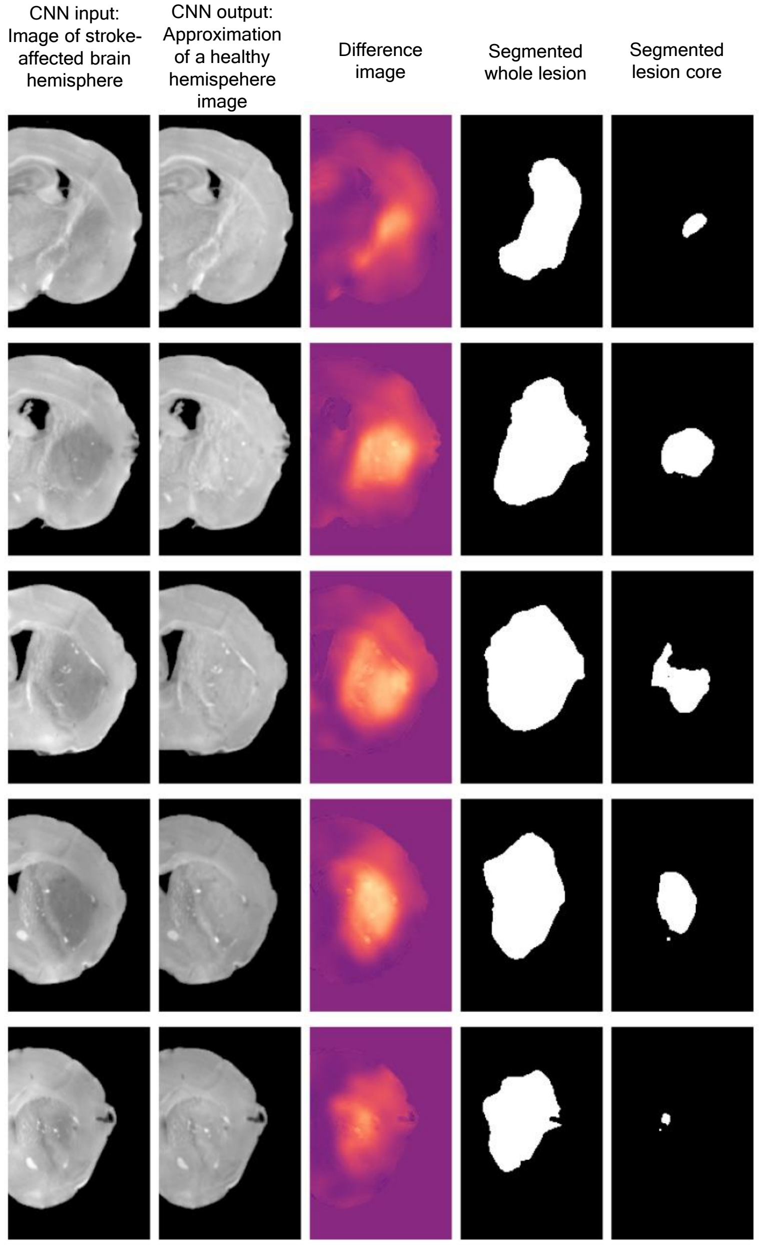

Supplement: Supplementary file 1 — Supplementary Information 1. [file 41598_2022_21494_MOESM1_ESM.pdf]
