# Supplementary material for: High-resolution micro-CT for 3D infarct characterization and segmentation in mice stroke models
Source: Sci Rep. 2022 Oct 19;12:17471. doi: 10.1038/s41598-022-21494-9 (PMC9582034; doi:10.1038/s41598-022-21494-9)
Supplement: Supplementary file 2 — Supplementary Information 2. [file 41598_2022_21494_MOESM2_ESM.docx]

**Supplemental material:**

High-resolution micro-CT for 3D infarct characterization and segmentation in mice stroke models

Raquel Pinto ^1,2*^, Jan Matula^5*^, Maria Gomez-Lazaro ^1,3^, Mafalda Sousa ^5^, Andrea Lobo ^6^ , Tomas Zikmund^5^, Jozef Kaiser ^5^, João R. Gomes ^1,2^

**Supplementary figure legends:**

**Suppl. Fig. 1- High-resolution micro-CT imaging of mice brains subjected to the stroke model (tMCAO 45 min) using iodine staining. (A)** Virtual slices (~4µm voxels) at different orientations from a micro-CT scan of a stroke mouse brain stained by inorganic iodine, showing total ischemic lesion, and core (orange/darker) and penumbra ((yellow/grey around the core) differentiation. The below panel shows lesion detail in the transaxial and sagittal planes. Scale bar: 2 mm.

**Suppl. Fig. 2- Iohexol, phosphotungstic acid (PTA), and phosphomolybdic acid (PMA) staining of whole mice brain.** Representative images of iohexol **(A)** and PTA **(B)** stained whole mice brains of mice subjected to tMCAO for 45 min following 24 h reperfusion, and PMA **(C)** stained mice brains not subjected to surgery. Iohexol does not confer contrast enhancement to brain structures, whereas PTA and PMA do not penetrate the whole sample/tissue.

**Suppl. Fig. 3- Sequential immunostaining process in iodine-stained mice brains. (A)** Representative 3D visualization of iodine-staining whole mouse brain volume (darker red), in relation to its volume before fixation and staining. Calculated volumes in CTAn demonstrated that the brain shrunk to a third of its size. **(B)** Images representing an iodine-stained tMCAO brain sliced after micro-CT scan, and labeled with the specific neuronal marker (NeuN) and a nuclear marker (Hoechst 33342). Fluorescent specific labeling was distinct between infarct lesions and healthy tissue.

**Suppl. Fig. 4- High-resolution micro-CT imaging of mice brains subjected to the TIA mouse model using Osmium Tetroxide staining. (A)** Virtual slices (~4µm voxels) at different orientations from micro-CT scan of a TIA brain stained with osmium tetroxide, showing degeneration of striatum white fibers (indicated by the white dotted surrounded region). The below panel shows lesion detail in the transaxial and sagittal planes. Scale bar: 2mm.

**Suppl. Fig. 5- Brain ischemic lesions (stroke and TIA models) progression using high-resolution micro-CT.** Representative images of sham, TIA, and stroke mice models brains using micro-CT imaging. Sham brain hemispheres mirror each other and demonstrate no ischemic alteration. TIA-subjected mice display striatum white matter degeneration. In the stroke model (tMCAO 45 min), we observe cortical and striatal lesions, and distinguish core and penumbra at 24 h following reperfusion. The stroke mouse brain model shows an increase in lesion size over the analyzed 3 to 72 hours, and 7 days post-reperfusion.

**Supplemental Fig. 6 - Neural network training and results visualization.** In **(A)** the mean training loss (MSE) in each training epoch is plotted. In **(B)** the trained CNN is applied on real lesion brain hemispheres and the segmentation of the whole lesion and lesion core is visualized.

**Suppl. Videos:**

**Suppl. Vid. 1- 3D whole brain of mouse stroke model (tMCAO 45 min) stained with osmium tetroxide and micro-CT imaged , using CTvox Bruker software.** The whole sample attenuation coefficient can be manipulated in the CTvox software to represent the whole brain in autonomous RGB scales, allowing further contrast of the anatomic features of interest. Therefore, the penumbra and core can be highlighted in the 3D context during stroke brain manipulation.

**Suppl. Vid. 2- 3D whole brain of TIA mouse model (tMCAO 10 min) stained with osmium tetroxide and micro-CT imaged, using CTvox Bruker software.** The whole sample attenuation coefficient can be manipulated in the CTvox software to represent the whole brain in autonomous RGB scales, allowing further contrast to the anatomic features of interest. Therefore, differences in striatum white fibers between hemispheres can be highlighted (blue) in the 3D context during brain manipulation, with the ipsilateral striatum demonstrating white fiber degeneration when compared to the contralateral side. Methodology is described in the manuscript and visually described.

**Suppl. Vid. 3- Visualization of the segmentation of the whole brain of a mouse stroke model (Avizo 2020.2, Thermo Fisher Scientific).** The methodology is described in the manuscript.

**Supplemental Methods:**

**Iohexol staining**

After tMCAO (45min- 24h), mice were transcardially perfused with 40 mL PBS, followed by 40 mL 4% paraformaldehyde (PFA) in PBS. Brains were carefully dissected and post-fixed in 4% PFA, at 4°C, for 1 week. Brains were then rinsed in PBS, and immersed in iohexol (GE Heathcare, Omnipaque 300mg/ml (iodine), 712498.6) diluted 1:2 in PBS (10ml total volume) ^1^, for 5 days at room temperature, under agitation and protected from light, before micro-CT imaging. As previously described ^2^, brains were wrapped in parafilm, to prevent brain dehydration, and scanned using Bruker SkyScan 1276 microCT scanner (Bruker, Belgium). Brain images shown in **Supplementary Fig. 1** were acquired using the following settings: 63 Kv and 200µA, 15µm spatial resolution, 0.4° rotation step through 180°, generating around 514 projections. An aluminium filter of 0.5mm was used, together with a frame averaging of 4.

**Phosphomolybdic acid staining:**

CD1 WT mice were transcardially perfused with PBS and 4% PFA, as previously described, and stained with 2,5% phosphmolybdenic acid (PMA) (Sigma-Aldrich, 221856) diluted in either demineralized 0,9% NaCl (**Suppl. Fig. 4**) or PBS (**Suppl. Fig. 3**) (as described here ^3^). Brains were immersed for 7 days, at 4°C, under agitation and protected by light. Brains were prepared for scanning as previously described and scanned with the following settings: 90 KV and 47 µA, 4 µm spatial resolution, 0.2º rotation step through 180 degrees, giving rise to 1801 projections. An aluminium filter of 1 mm was used, together with a frame averaging of 4. It was clear that PMA only penetrated mouse brain with PBS as solvent. However, the detail of the brain mouse structures is noisier than from the osmium/iodine stain.

**Phosphotungstic acid staining:**

After tMCAO (45min- 24h), mice were transcardially perfused with PBS and PFA, as previously described, and stained with 2.5% phosphotungstic acid (PTA) (Sigma, Ht152) diluted in demineralized water as previously described ^4^ for 5 days (10mL total volume), at 4°C, under agitation and protected from light. The brain images shown in **Supplementary Fig. 2** were acquired using the following settings: 90KV and 47µA, 8µm spatial resolution, 0.4° rotation step through 180°, originating around 514 projections. An aluminium filter of 1mm was used, together with a frame averaging of 4.

**Bibliography:**

1. Kuts R, Frank D, Gruenbaum BF, et al. A Novel Method for Assessing Cerebral Edema, Infarcted Zone and Blood-Brain Barrier Breakdown in a Single Post-stroke Rodent Brain. *Frontiers in neuroscience* 2019; 13: 1105-1105. DOI: 10.3389/fnins.2019.01105.

2. Dobrivojevic M, Bohacek I, Erjavec I, et al. Computed microtomography visualization and quantification of mouse ischemic brain lesion by nonionic radio contrast agents. *Croat Med J* 2013; 54: 3-11. 2013/02/28. DOI: 10.3325/cmj.2013.54.3.

3. Dobrivojevic M, Spiranec K, Gorup D, et al. Urodilatin reverses the detrimental influence of bradykinin in acute ischemic stroke. *Exp Neurol* 2016; 284: 1-10. 2016/07/20. DOI: 10.1016/j.expneurol.2016.07.007.

4. Descamps E, Sochacka A, De Kegel B, et al. Soft tissue discrimination with contrast agents using micro-CT scanning. *Belg J Zool* 2014; 144: 20-40.
